# Label-free quantitative proteomics of arbuscular mycorrhizal *Elaeagnus angustifolia* seedlings provides insights into salt-stress tolerance mechanisms

**DOI:** 10.3389/fpls.2022.1098260

**Published:** 2023-01-10

**Authors:** Wei Chang, Yan Zhang, Yuan Ping, Kun Li, Dan-Dan Qi, Fu-Qiang Song

**Affiliations:** ^1^ Heilongjiang Provincial Key Laboratory of Ecological Restoration and Resource Utilization for Cold Region, School of Life Sciences, Heilongjiang University, Harbin, China; ^2^ Jiaxiang Industrial Technology Research Institute of Heilongjiang University, Jinin, China

**Keywords:** arbuscular mycorrhizal fungi (AM fungi*)*, *Elaeagnus angustifolia*, salt stress, proteomics, *Rhizophagus irregularis*

## Abstract

**Introduction:**

Soil salinization has become one of the most serious environmental issues globally. Excessive accumulation of soluble salts will adversely affect the survival, growth, and reproduction of plants. Elaeagnus angustifolia L., commonly known as oleaster or Russian olive, has the characteristics of tolerance to drought and salt. Arbuscular mycorrhizal (AM) fungi are considered to be bio-ameliorator of saline soils that can enhance the salt tolerance of the host plants. However, there is little information on the root proteomics of AM plants under salt stress.

**Methods:**

In this study, a label-free quantitative proteomics method was employed to identify the differentially abundant proteins in AM E. angustifolia seedlings under salt stress.

**Results:**

The results showed that a total of 170 proteins were significantly differentially regulated in E.angustifolia seedlings after AMF inoculation under salt stress. Mycorrhizal symbiosis helps the host plant E. angustifolia to respond positively to salt stress and enhances its salt tolerance by regulating the activities of some key proteins related to amino acid metabolism, lipid metabolism, and glutathione metabolism in root tissues.

**Conclusion:**

Aspartate aminotransferase, dehydratase-enolase-phosphatase 1 (DEP1), phospholipases D, diacylglycerol kinase, glycerol-3-phosphate O-acyltransferases, and gamma-glutamyl transpeptidases may play important roles in mitigating the detrimental effect of salt stress on mycorrhizal E. angustifolia . In conclusion, these findings provide new insights into the salt-stress tolerance mechanisms of AM E. angustifolia seedlings and also clarify the role of AM fungi in the molecular regulation network of E. angustifolia under salt stress.

## 1 Introduction

Soil salinization is one of the most serious global environmental problems, which changes soil physical and chemical properties, affects plant growth and development, and reduces crop yield (Zelm et al., 2020). The detrimental effects of salt stress on plants mainly include three aspects: (1) sodium accumulation in the soil reduces water availability and causes physiological drought in plants (Dastogeer et al., 2020); (2) excess sodium and chlorine ions causes ion imbalances, which affects the absorption of mineral ions by plants and inhibits photosynthesis (Santander et al., 2019); (3) the toxic effects of sodium and chlorine ions induces excessive production of reactive oxygen species (ROS), which results in lipid peroxidation and oxidative damage to proteins and nucleic acids (Alqarawi et al., 2014). To adapt to a salinized environment, some plants, especially halophytes, have developed strategies to counter salt stress, such as accumulation of compatible osmolytes, maintenance of ion homeostasis, regulation of water uptake, enhancement of photosynthesis, detoxification of ROS, and induction of phytohormones (Evelin et al., 2019). Bioremediation of saline soil using halophytes has gained attention as a cost-effective and environmentally friendly remediation technology.

Arbuscular mycorrhizal (AM) fungi, belonging to the Glomeromycota, are obligate biotrophs that can establish symbiotic associations with the roots of approximately 80% terrestrial plant species (Lin et al., 2017; Chandrasekaran et al., 2021). AM symbiosis can augment the host plant’s ability to uptake and transfer water and mineral nutrients from the soil through a large amount of extramatrical hyphae; hence, it is conducive to plant growth and enhances the abiotic stress tolerance of plants. At present, accumulating evidence has shown that AM symbiosis can help improve plant tolerance to salt stress *via* various physiological and biochemical mechanisms (Duc et al., 2021). For instance, AM symbiosis can augment water-use eficiency, improve nutrient acquisition, maintain ionic homeostasis and osmotic balance, preserve cell ultrastructure, stimulate antioxidant activities to prevent damage due to excess ROS, enhance photosynthetic efficiency, and regulate phytohormone levels to overcome the deleterious effects of salts on plant growth and development (Evelin et al., 2019). In addition, several studies on various plant species have been conducted to elucidate the molecular mechanisms of salt tolerance in AM plants. For example, AM fungi control the expression of aquaporin genes and presumably regulate water flow in tomato under salt stress (Ouziad et al., 2006). The high concentration of N in AM plants under salt stress is due to increased expression of nitrate and ammonium transporters, as demonstrated in wheat (Veronica et al., 2017). Colonization by AM fungi can upregulate the expression of three chloroplast genes (*RppsbA*, *RppsbD*, and *RprbcL*) in the leaves as well as three genes (*RpSOS1*, *RpHKT1*, and *RpSKOR*) encoding membrane transport proteins involved in K^+^/Na^+^ homeostasis in the roots of black locust under salt stress (Jie et al., 2017). AM symbiosis significantly upregulated the expression of *OsNHX3, OsSOS1*, *OsHKT2;1*, and *OsHKT1;*5 in rice plants under saline conditions (Porcel et al., 2016). Exploring the underlying molecular mechanisms involved is an essential step in understanding the salt tolerance induced by AM fungi, but such studies are limited. To date, little is known about the molecular mechanisms of AM fungi in enhancing the ability of plants to resist salt stress.

*Elaeagnus angustifolia* L., a member of the Elaeagnaceae family, is an halophyte and deciduous plant with small and reddish brown fruits (Mao et al., 2022). Many studies have focused on the utilization of *E. angustifolia* fruits, flowers, and plant extracts owing to the considerable nutritional value and remedial applications (Safdari and Khadivi, 2021). As this plant can grow in a wide range of adverse environmental conditions, such as severe drought, rocky soil, and saline soil (Safdari and Khadivi, 2021; Guo and Wang, 2021), it is widely used for urban and road greening, sand fixation, and saline soil improvement. Moreover, *E. angustifolia* can tolerate a high concentration of salt while maintaining its growth (Guo and Wang, 2021). Some studies show that AM fungi can enhance salt resistance in halophytes (Dashtebani et al., 2014; Zhang et al., 2014). Previous studies have demonstrated that inoculation of AM fungi can ameliorate the negative effects of salt stress on *E. angustifolia* (Chang et al., 2018; Liang et al., 2021). A recent meta-analysis indicates that halophytes and glycophytes have different physiological responses to AM fungi under salt conditions (Pan et al., 2020). AM fungi can promote halophytes growth under salt stress, mainly through the efficient and effective use of inorganic ions and organic osmolytes (Diao et al., 2021). Unlike halophytes, AM fungi can mitigate the damage from salt stress in glycophytes, which is mainly due to the regulations of accumulating soluble sugar, improving nutrients acquisition, reducing sodium ion accumulation, enhancing super-oxide dismutase activity, and elevating chlorophyll synthesis (Diao et al., 2021). These differences may be attributed to the inherent molecular mechanisms of salt tolerance in glycophytes and halophytes.

Moreover, investigation of the proteome-wide response of *E. angustifolia* leaves suggested that AM fungi increased the secondary metabolism level of the phenylpropane pathway, enhanced the signal transduction of Ca^2+^ and scavenging of ROS, promoted the biosynthesis of protein and ATP, accelerated the protein folding, and inhibited the degradation of protein under salt stress (Jia et al., 2019). Root tissues, which directly form symbiotic structures with AM fungi, are crucial for regulating the uptake of water and nutrients and enhancing plant resistance to salt stress (Evelin et al., 2019; Zhang et al., 2021). However, there is little information on the molecular mechanisms between plant roots and AM fungi in mitigating salt stress. Label-free quantitative (LFQ) proteomics is an effective method for studying the proteome, which is helpful to explore the molecular mechanism of abiotic stresses tolerance. In the past few years, LFQ proteomic analysis have been applied to reveal the response mechanisms of plant roots to abiotic stresses, including flooding, drought, heavy metal, and low N (Nanjo et al., 2013; Xin et al., 2016; Jozefowicz et al., 2017; Borges et al., 2019; Pinto et al., 2021).

Therefore, in this study, we performed the first AM proteomic analysis of *E. angustifolia* seedling roots under different salt stress using an LFQ proteomic approach. This work aimed to reveal the AM proteome profile of *E. angustifolia* and the key AM proteins involved in salt stress response. Our results would provide novel insights to the underlying molecular mechanisms of salt tolerance induced by AM fungi in plants.

## 2 Materials and methods

### 2.1 Plant materials

Seeds of *E. angustifolia* were provided by Heilongjiang Jinxiudadi Biological Engineering Co., Ltd. (Haerbin, China). After surface sterilization in 0.2% KMnO_4_ solution for 10 min and rinsing four times with sterile distilled water, the seeds were grown in plastic pots (5 L) containing a substrate (soil:vermiculite = 3:1, V/V), previously sterilized in an autoclave for 1 h at 121°C three times on alternate days (Chang et al., 2018). The soil was collected from the Forest Botanical Garden of Heilongjiang Province (China, 45° 42′ 40.09″ N 126° 38′ 22.23″ W), sieved (5 mm), and diluted with vermiculite (soil:vermiculite = 3:1, V/V) (Chang et al., 2018).

### 2.2 Experimental design

The experiment was conducted in a randomized complete block design with four treatments: (1) non-inoculated *E. angustifolia* without salt stress (NM0); (2) non-inoculated *E. angustifolia* under salt stress conditions (300 mmol/LNaCl) (NM300); (3) inoculated *E. angustifolia* without salt stress (M0); and (4) inoculated *E. angustifolia* under salt stress conditions (300 mmol/LNaCl) (M300). Each treatment had eight replicates (pots). The experiment was carried out under natural outdoor conditions. The positions of the pots were changed each week.

### 2.3 Inoculation and salt stress treatments

The AM fungus species used in the study was *Rhizophagus irregularis* previously isolated from the Zhao YueShan National Wetland Park (Heilongjiang Province, China). The mycorrhizal inoculum contained approximately 25–30 AM propagules per gram (Chang et al., 2018). The soil was inoculated with *R. irregularis* at the time of sowing (Chang et al., 2018). The inoculated dosage of mycorrhizal inoculum per pot was 10 g (Chang et al., 2018). The same amount of autoclaved mycorrhizal inoculum was used in non-inoculated treatments.

After approximately 100 days of inoculation with the mycorrhizal inoculum, saline solution was added into the pots (Chang et al., 2018). To avoid osmotic shock, NaCl concentration in the soil was increased gradually by adding 1.5L 2 mol/L stock saline solution to each pot on alternate days (Chang et al., 2018). It took 6 days to achieve the desired levels of 300 mmol/L NaCl. The seedlings continued to be cultivated for 30 days (Chang et al., 2018). Six pots of *E. angustifolia* seedlings with relatively consistent growth were selected from NM0, NM 300, M0, and M300 treatments respectively, and three seedlings were randomly selected from each pot. The root of each seedling was taken and then quickly washed with sterile water. Roots from two pots of the same treatment were combined as one replicate. There were three biological repeats per treatment. The proteomic profile and AM colonization of the samples were detected.

### 2.4 Mycorrhizal colonization

The fine roots were cut into 1-cm segments, cleared in 10% KOH, and stained with 0.05% trypan blue. Thirty fragments were examined for AM colonization under a digital computerized microscope (Model DP-73; Olympus, JPN). All AM fungal structures including spore, hyphae, arbuscules, and vesicles found in the roots were recorded. The mycorrhizal percentage was determined by the method of McGonigle (Mcgonigle et al., 1990).

### 2.5 Extraction and quantification of proteins

The roots of all samples were ground into fine powder using a mortar in liquid nitrogen. Total protein was extracted from the roots using the method of Jia et al. (2019) and quantified using a BCA Protein Assay Kit (P0012; Beyotime, Shanghai, China).

### 2.6 Filter-aided sample preparation digestion

Three hundred micrograms of proteins for each sample was mixed with 100 mM DTT. The mixture was placed in a boiling water bath for 5 min and then cooled to 25°C. Next, 200 µL of UA buffer (8 M Urea, 150 mM Tris-HCl pH 8.0) was incorporated into the mixture. The mixture was then filtered using a ultrafiltration centrifugal tube (30 kD) at 14,000 × *g* for 15 min. After that, the filters were washed with 200 µL of UA buffer by centrifugation for 15 min at 14,000 × *g*, and the filtrate was discarded. Next, 100 µL of iodoacetamide (50 mM IAA in UA buffer) was added into each tube. The mixture was agitated for 1 min at 600 rpm, incubated for 30 min at 25°C in darkness, and then centrifuged for 10 min at 14,000 × *g*. The filters were washed with 100 µL of UA buffer three times and subsequently with 100 µL of 25 mM NH_4_HCO_3_ buffer twice, followed by centrifugation for 10 min at 14,000 × *g*. Finally, 40 µL of trypsin buffer (2 µg trypsin in 40 µL of 50 mM NH_4_HCO_3_) was added, and the mixture was shaken for 1 min at 600 rpm and incubated for 16–18 h at 37°C. The digested sample was transferred to a new collection tube. After centrifugation for 10 min at 14,000 × *g*, the filtrate was collected, desalted with C18-SD Extraction Disk Cartridge (3 M, USA), and quantified by measuring the excitation at 280 nm.

### 2.7 LC-MS/MS analysis of enzymatic hydrolysates

The products were analyzed after enzymatic hydrolysis using LC-MS/MS. Each sample was separated by using an Easy nLC 1000 liquid phase system (Thermo, USA). The mobile phase consisted of buffer A [0.1% formic acid acetonitrile solution (2% acetonitrile)] and buffer B (84% acetonitrile and 0.1% formic acid). The chromatographic column was balanced with 100% A solution. The sample was loaded onto the column (Thermo EASY column SC001traps 150 μm × 20 mm, RP-C18) by an automatic sampler and separated by an analytical column at a flow rate of 400 nL/min. The gradient elution procedure was as follows: 0–100 min, 0–45% buffer B; 100–108 min, 45–100% buffer B; and 108–120 min, 100% buffer B. After capillary separation using a capillary high-performance liquid chromatography system, the protein-digested products were analyzed using a Q-Exactive mass spectrometer (Thermo Finnigan, USA), with the following settings: duration: 120 min; detection method: positive ion detection; parent ion scanning scope, 300–1800 *m/z*; mass-charge ratios of polypeptide and polypeptide fragments collection method, 20 debris maps (MS^2^ scan, HCD); MS^1^ resolution at M/Z 200, 70,000; and MS^2^ resolution at M/Z 200, 17,500.

### 2.8 Database search and protein identification and quantitation

The 12 LC-MS/MS RAW files were imported into Maxquant 1.3.0.5 (Wiśniewski et al., 2009) for database retrieval and LFQ analysis. The database used was P16440_Unigene.fasta.transdecoder_73797_20161212.fasta (Sequence73797, self-building). The maxquant software parameter table is shown in **Table 1**. Search results were analyzed with the Perseus software platform (version 1.3.0.4). Based on the proteins identified by mass spectrometry in the RAW files, differentially expressed proteins (DEPs) were screened according to the screening criteria of ratio > +/−2 and *p* value < 0.05. The quantified protein sequence information was extracted from the UniProtKB database (version number: 201701).

**Table 1:** Database retrieval parameter table.

Item	Value
Main search ppm	6
Missed cleavage	2
MS/MS tolerance ppm	20
De-Isotopic	TRUE
enzyme	Trypsin
database	P16440_Unigene.fasta.transdecoder_73797_20161212.fasta
Fixed modification	Carbamidomethyl (C)
Variable modification	Oxidation(M), Acetyl (Protein N-term)
Decoy database pattern	reverse
LFQ	TRUE
LFQ min. ratio count	1
Match between runs	2min
Peptide FDR	0.01
Protein FDR	0.01

### 2.9 Protein GO annotation, KEGG pathway annotation, and functional enrichment analysis

GO annotation of target protein sets was performed using the Blast2GO software (version 3.3.5) (Ashburner et al., 2000; Quevillon et al., 2005; Götz et al., 2008). The KEGG Automatic Annotation Server (KASS) software was used for KEGG pathway annotation (Kanehisa et al., 2011). GO enrichment on three ontologies (biological process, molecular function, and cellular component) and KEGG pathway enrichment analysis were applied based on Fisher’s exact test, considering the whole quantified proteins annotation as a background dataset. Benjamini–Hochberg correction for multiple testing was further applied to adjust the derived p-values. Only functional categories and pathways with *p*-values under a threshold of 0.05 were considered significant. Principal component analysis (PCA) was performed using RStudio (version 1.2.5042) with the packages FactoMineR and Factoextra. STRING database version 11.5 was used for the protein-protein interaction (PPI) analysis. Cytoscape version 3.9.1 was used for visual representation.

## 3 Results

### 3.1 Growth of *Elaeagnus angustifolia* under salt stress

**Figure 1:**
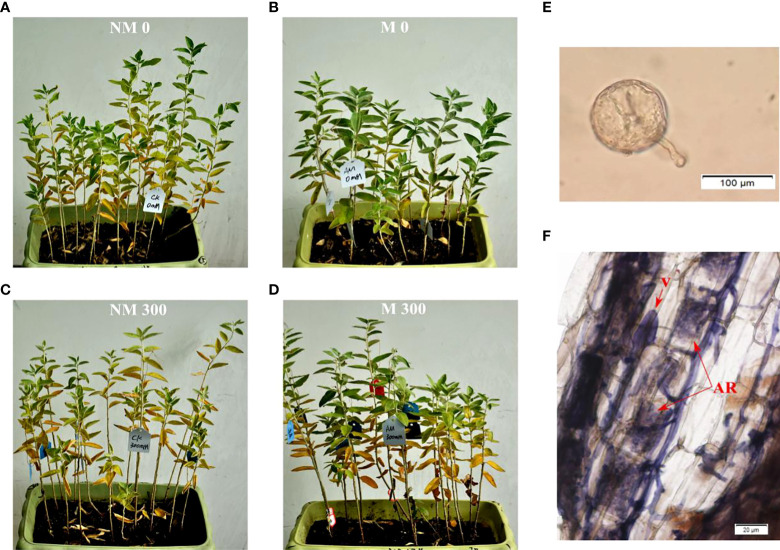
Growth of *E. angustifolia* inoculated AM fungi under salt stress. **(A)** NM0: non-mycorrhizal, 0 mmol/L NaCl; **(B)** M0: mycorrhizal, 0 mmol/L NaCl; **(C)** NM300: non-mycorrhizal, 300 mmol/L NaCl; **(D)** M300: mycorrhizal, 300 mmol/L NaCl. **(E)** Photomicrographs of AM fungi spore; **(F)** Photomicrographs of structural colonization of AM fungi in the roots of *E. angustifolia*. Vesicles (V); Arbuscule (AR).

Mycorrhizal seedlings had a greater height, diameter, length, and area than non-mycorrhizal seedlings under salt stress in previous studies (Chang et al., 2018; Jia et al., 2019; He et al., 2019; Liang et al., 2021). As shown in **Figures 1A, B**, both mycorrhizal and non-mycorrhizal seedlings grew well in the treatments lacking salt, but the leaves of the mycorrhizal seedlings were stronger than those of non-mycorrhizal seedlings. Some leaves of the mycorrhizal and non-mycorrhizal seedlings were yellow under salt stress (**Figures 1C, D**); however, the number of withered leaves of mycorrhizal plants was significantly lower than that of non-mycorrhizal plants (**Figures 1C, D**).

### 3.2 Colonization of AM fungi in plant roots

The typical morphological structures of AM fungi were detected in inoculated *E. angustifolia* fine roots, including the spores, vesicles and arbuscules (**Figures 1E, F** **)**. The maximum AM colonization percentage of the root was 96% at approximately 100 days after inoculation. This high rate of AM colonization indicated that *E. angustifolia* and *R. irregularis* established a vigorous symbiosis before the salt stress treatment. No colonization was found in the non-inoculated seedlings.

### 3.3 Protein identification and comparison

**Figure 2:**
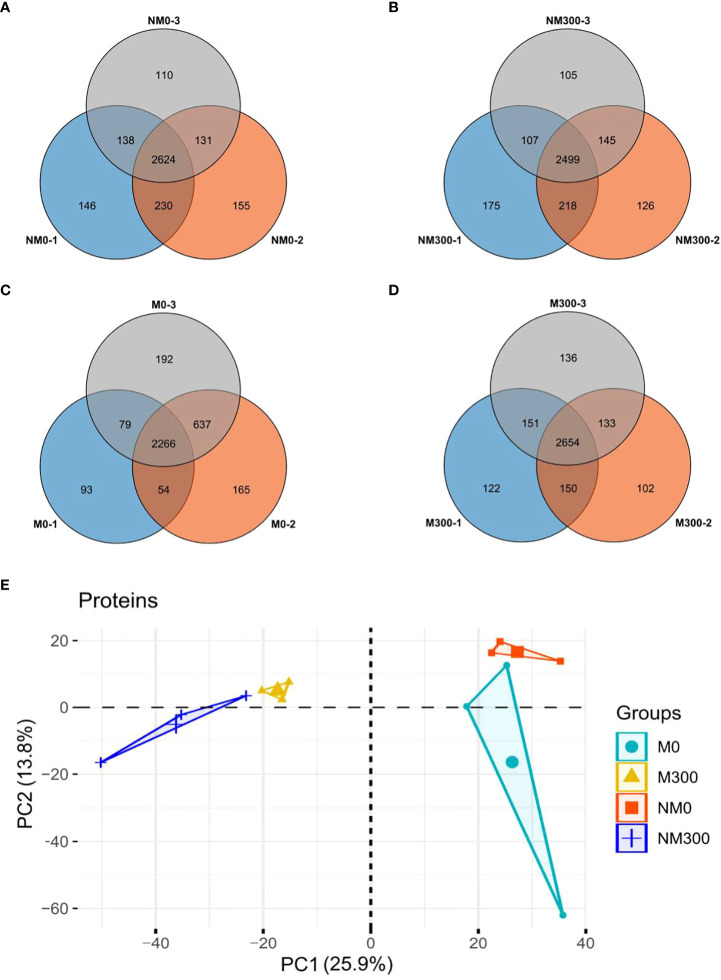
Statistics of the number of proteins identified in each treatment group. **(A)** Venn diagram of the number of proteins identified in the NM0 group; **(B)** Venn diagram of the number of proteins identified in the NM300 group; **(C)** Venn diagram of the number of proteins identified in the M0 group; **(D)** Venn diagram of the number of proteins identified in the M300 group; **(E)** PCA of proteomic data in each treatment group.

In the four treatment groups, a total of 22,681 peptides were identified, matching 4227 proteins from *E. angustifolia* roots. The number of proteins identified in the three replicates of each treatment group is shown in **Figures 2A-D**. Quantifiable proteins were identified in at least two of the three replicates for further analysis. To estimate the variability of the obtained proteomic data, we assessed clustering of the observed data using PCA. The PCA plot (**Figure 2E**) indicated difference among treatment groups, and at the global proteomic level, distinct separation of the NM0 and M0 from NM300 and M300. The significance of the difference in protein abundance was filtered by ratio > ± 2 and *p* value < 0.05. A total of 502 (NM0 vs. NM300), 157 (NM0 vs. M0), 340 (M300 vs. M0), and 137 (M300 vs. NM300) differentially abundant proteins (DAPs) were identified among the four groups (**Table 2**).

**Table 2:** Differentially abundant proteins (DAPs) between treatment groups.

Treatments	Total Number of DAPs	The Number of DAPs Annotated with GO Term	The Number of DAPs Annotated in the KEGG database
NM0 vs NM300	502	331	76
NM0 vs M0	157	107	11
M300 vs M0	340	242	28
M300 vs NM300	137	58	13

### 3.4 Functional enrichment analysis of DAPs

Gene Ontology (GO) and Kyoto Encyclopedia of Genes and Genomes (KEGG) pathway enrichment analysis (Fisher’s exact test, *p*<0.05) of DAPs were performed to explore the potential function of the DAPs identified in this study. As shown in **Table 2**, a total of 331 and 76 (NM0 vs. NM300), 107 and 11 (NM0 vs. M0), 242 and 28 (M300 vs. M0), and 58 and 13 (M300 vs. NM300) DAPs were annotated in the GO and KEGG databases, respectively.

**Figure 3:**
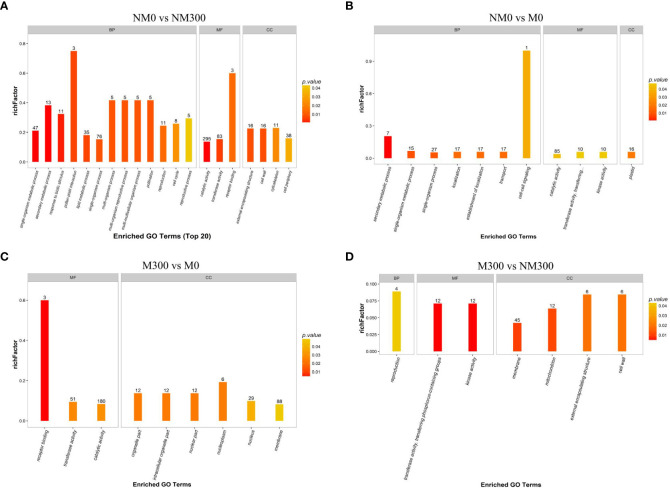
Gene Ontology (GO) enrichment analysis of the differentially abundant proteins (DAPs) in each comparison group. **(A)** GO categories of DAPs in NM0 vs. NM300; **(B)** GO categories of DAPs in NM0 vs. M0; **(C)** GO categories of DAPs in M300 vs. M0; **(D)** GO categories of DAPs in M300 vs. NM300. Abbreviations: BP, Biological processes; MF, Molecular functions; CC, Cellular components.

GO enrichment analysis of DAPs was performed to classify the biological processes, molecular functions, and cellular components, as presented in **Figure 3**. The DAPs between the NM0 vs. NM300 groups were mainly enriched in catalytic activity (GO: 0003824), transferase activity (GO:0016740), single-organism process (GO:0044699), single-organism metabolic process (GO:0044710), and lipid metabolic process (GO:0006629) (**Figure 3A**). The DAPs between NM0 vs. M0 were mainly enriched in catalytic activity (GO: 0003824), single-organism process (GO:0044699), establishment of localization (GO:0051234), transport (GO:0006810), localization (GO:0051179), plastid (GO:0009536), and single-organism metabolic process (GO:0044710) (**Figure 3B**). The DAPs between M300 vs. M0 were mainly enriched in catalytic activity (GO: 0003824), membrane (GO:0016020), transferase activity (GO:0016740), and nucleus (GO:0005634) (**Figure 3C**). The DAPs between M300 vs. NM300 were mainly enriched in membrane (GO:0016020), kinase activity (GO:0016301), mitochondrion (GO:0005739), and transferase activity, transferring phosphorus-containing groups (GO:0016772) (**Figure 3D**).

**Figure 4:**
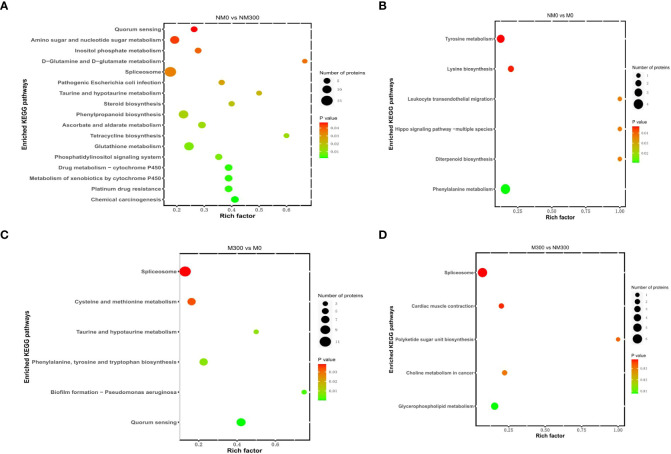
Kyoto Encyclopedia of Genes and Genomes (KEGG) pathway enrichment analysis of DAPs in each comparison group. **(A)** KEGG pathways of DAPs in NM0 vs. NM300; **(B)** KEGG pathways of DAPs in NM0 vs. M0; **(C)** KEGG pathways of DAPs in M300 vs. M0; **(D)** KEGG pathways of DAPs in M300 vs. NM300.

The KEGG pathway enrichment results are shown in **Figure 4**. For NM0 vs. NM300, the DAPs were significantly enriched in 17 metabolic pathways (**Figure 4A**), such as spliceosome (map03040), phenylpropanoid biosynthesis (map00940), glutathione metabolism (map00480), amino sugar and nucleotide sugar metabolism (map00520), ascorbate and aldarate metabolism (map00053), drug metabolism-cytochrome P450 (map00982), metabolism of xenobiotics by cytochrome P450 (map00980), phosphatidylinositol signaling system (map04070), and inositol phosphate metabolism (map00562). For NM0 vs. M0, the DAPs were significantly enriched in six metabolic pathways (**Figure 4B**), namely phenylalanine metabolism (map00360), tyrosine metabolism (map00350), lysine biosynthesis (map00300), leukocyte transendothelial migration (map04670), diterpenoid biosynthesis (map00904), and Hippo signaling pathway-multiple species (map04392). For M300 vs. M0, the DAPs were also significantly enriched in six metabolic pathways (**Figure 4C**), that is, spliceosome (map03040); quorum sensing (map02024); cysteine and methionine metabolism (map00270); phenylalanine, tyrosine, and tryptophan biosynthesis (map00400); taurine and hypotaurine metabolism (map00430); and biofilm formation – *Pseudomonas aeruginosa* (map02025). For M300 vs. NM300, the DAPs were significantly enriched in five metabolic pathways (**Figure 4D**), including spliceosome (map03040), glycerophospholipid metabolism (map00564), choline metabolism in cancer (map05231), cardiac muscle contraction (map04260), and polyketide sugar unit biosynthesis (map00523).

### 3.5 Screening of root salt tolerance-related DAPs in mycorrhizal *Elaeagnus angustifolia* seedlings

**Figure 5:**
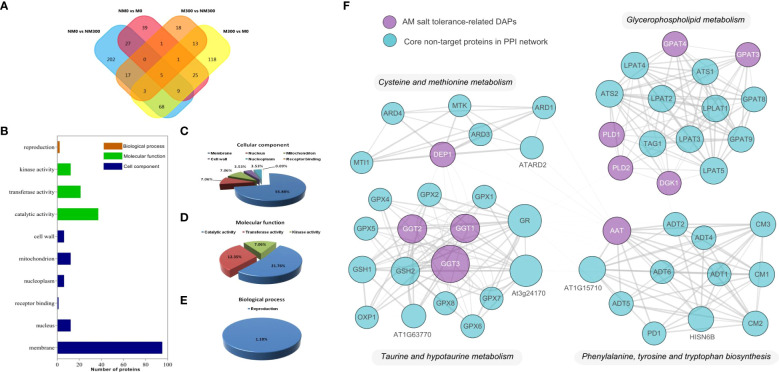
Proteomic analysis of 170 roots’ salt tolerance-related DAPs in Mycorrhizal *E. angustifolia* Seedlings. **(A)** Venn diagram of the DAPs between the NM0 vs. NM300, NM0 vs. M0, M300 vs. M0 and M300 vs. NM300 comparison group; **(B)** Biological functional classification of the 170 DAPs; **(C)** Distribution of the 113 DAPs in terms of cellular component; **(D)** Distribution of the 57 DAPs in terms of molecular function; **(E)** Distribution of the 57 DAPs in terms of biological process; **(F)** The protein-protein interactions (PPI) between the AM salt tolerance-related DAPs. Abbreviations: AAT, aspartate aminotransferase; DEP1, dehydratase-enolase-phosphatase 1; PLD, phospholipase D; DGK, diacylglycerol kinase; GPAT, glycerol-3-phosphate O-acyltransferase; GGT, gamma-glutamyl transpeptidase.

To understand the molecular mechanism of *R. irregularis* in mitigating the damage of NaCl stress, DAPs related to root salt tolerance were screened. As shown in **Figure 5A**, after comparing DAPs among the four groups (NM0 vs. NM300, NM0 vs. M0, M300 vs. M0, and M300 vs. NM300) and removing overlapping DAPs, we finally screened and determined 170 DAPs related to salt tolerance in the roots of mycorrhizal *E. angustifolia* seedlings based on the GO enrichment analysis results. These DAPs comprised 129 DAPs between M300 vs. M0 and 41 DAPs between M300 vs. NM300, including 22 upregulated proteins, 29 downregulated proteins, 50 unexpressed proteins, and 69 newly emerged proteins (**Supplementary Table 1**).

Based on the GO enrichment results of M300 vs. M0 and M300 vs. NM300, the 170 DAPs were classified to biological process, molecular function, and cellular component, as shown in **Figure 5B**. In terms of biological process, 2 of the 170 DAPs were addressed to terms of reproduction (GO:0000003) (**Figures 5B, E**). In the cellular component category, 113 of the 170 DAPs were associated with the membrane (GO:0016020), nucleus (GO:0005634), mitochondrion (GO:0005739), nucleoplasm (GO:0005654), cell wall (GO:0005739), and receptor binding (GO:0005102), among which DAPs associated with the cell membrane (84.07%) were the most frequent (**Figures 5B, C**). For molecular function, 57 of the 170 DAPs were involved in catalytic activity (GO:0003824), transferase activity (GO:0016740), and kinase activity (GO:0016301), among which DAPs related to catalytic activity (64.91%) were the most frequent (**Figures 5B, D**).

Proteins in organisms do not exert their biological functions independently but rather coordinate with each other to complete a series of biochemical reactions. Therefore, pathway analysis is helpful to systematically and comprehensively understand biological processes, characteristics, disease mechanisms and stress-resistance mechanisms in cells. As shown in **Table 3**, based on the GO enrichment analysis results of M300 vs. M0 and M300 vs. NM300, 20 of the 170 DAPs were mapped to nine KEGG metabolic pathways, including cysteine and methionine metabolism (map00270), phenylalanine, tyrosine, and tryptophan biosynthesis (map00400), taurine and hypotaurine metabolism (map00430), glycerophospholipid metabolism (map00564), choline metabolism in cancer (map05231), quorum sensing (map02024), biofilm formation – *P. aeruginosa* (map02025), spliceosome (map03040), and cardiac muscle contraction (map04260).

**Table 3:** The KEGG pathways of mycorrhizal symbiotic salt-tolerance related DAPs.

Map ID	Map Name	Protein ID	KO entry	Protein name	Difference expression	*p* value	FDR	Comprision group
map00270	Cysteine and methionine metabolism	TR79899|c1_g1_i1|m.44380	K14454	aspartate aminotransferase, cytoplasmic [EC:2.6.1.1];	Up-regulated	0.0336176	0.3842673	M300-M0
		TR105434|c2_g1_i2|m.66385	K16054	methylthioribulose 1-phosphate dehydratase/enolase-phosphatase E1 [EC:4.2.1.109;3.1.3.77];	Newly-expressed	0.0336176	0.3842673	M300-M0
		TR79846|c2_g2_i5|m.44255	K00789	S-adenosylmethionine synthetase [EC:2.5.1.6];	Down-regulated	0.0336176	0.3842673	M300-M0
		TR132629|c0_g2_i2|m.91692	K00928	aspartate kinase [EC:2.7.2.4];	Non-expressed	0.0336176	0.3842673	M300-M0
map00400	Phenylalanine, tyrosine and tryptophan biosynthesis	TR79899|c1_g1_i1|m.44380	K14454	aspartate aminotransferase, cytoplasmic [EC:2.6.1.1];	Up-regulated	0.0073897	0.3175952	M300-M0
		TR143362|c0_g1_i3|m.100535	K01657	anthranilate synthase component I [EC:4.1.3.27];	Down-regulated	0.0073897	0.3175952	M300-M0
		TR132556|c2_g1_i1|m.91459	K01626	3-deoxy-7-phosphoheptulonate synthase [EC:2.5.1.54];	Down-regulated	0.0073897	0.3175952	M300-M0
		TR26801|c0_g1_i1|m.12368	K01626	3-deoxy-7-phosphoheptulonate synthase [EC:2.5.1.54];	Non-expressed	0.0073897	0.3175952	M300-M0
map00430	Taurine and hypotaurine metabolism	TR63112|c0_g1_i1|m.30616	K18592	gamma-glutamyltranspeptidase/glutathione hydrolase/leukotriene-C4 hydrolase [EC:2.3.2.2;3.4.19.13;3.4.19.14];	Newly-expressed	0.0079898	0.3175952	M300-M0
map00564	Glycerophospholipid metabolism	TR79915|c2_g1_i5|m.44422	K13506	glycerol-3-phosphate O-acyltransferase 3/4 [EC:2.3.1.15];	Newly-expressed	0.0079694	0.3754118	M300-NM300
		TR109938|c0_g1_i2|m.71087	K01115	phospholipase D1/2 [EC:3.1.4.4];	Newly-expressed	0.0079694	0.3754118	M300-NM300
		TR138836|c1_g1_i2|m.95935	K00901	diacylglycerol kinase (ATP) [EC:2.7.1.107];	Newly-expressed	0.0079694	0.3754118	M300-NM300
		TR85343|c0_g1_i1|m.49167	K00111	glycerol-3-phosphate dehydrogenase [EC:1.1.5.3];	Non-expressed	0.0079694	0.3754118	M300-NM300
map02024	Quorum sensing	TR135538|c0_g1_i2|m.93124	K01897	long-chain acyl-CoA synthetase [EC:6.2.1.3];	Down-regulated	4.89E-05	0.0077706	M300-M0
		TR143362|c0_g1_i3|m.100535	K01657	anthranilate synthase component I [EC:4.1.3.27];	Down-regulated	4.89E-05	0.0077706	M300-M0
		TR132556|c2_g1_i1|m.91459	K01626	3-deoxy-7-phosphoheptulonate synthase [EC:2.5.1.54];	Down-regulated	4.89E-05	0.0077706	M300-M0
		TR26801|c0_g1_i1|m.12368	K01626	3-deoxy-7-phosphoheptulonate synthase [EC:2.5.1.54];	Non-expressed	4.89E-05	0.0077706	M300-M0
map02025	Biofilm formation - Pseudomonas aeruginosa	TR143362|c0_g1_i3|m.100535	K01657	anthranilate synthase component I [EC:4.1.3.27];	Down-regulated	0.0018863	0.1499648	M300-M0
map03040	Spliceosome	TR69588|c0_g1_i1|m.36222	K12890	splicing factor, arginine/serine-rich 1/9	Down-regulated	0.0383431	0.3842673	M300-M0
		TR92675|c0_g1_i2|m.55079	K12830	splicing factor 3B subunit 3	Down-regulated	0.0383431	0.3842673	M300-M0
		TR127978|c0_g1_i1|m.86394	K12869	crooked neck	Non-expressed	0.0383431	0.3842673	M300-M0
		TR98171|c0_g1_i1|m.59032	K12859	U5 snRNP protein, DIM1 family	Non-expressed	0.0383431	0.3842673	M300-M0
		TR143366|c1_g1_i1|m.100540	K06063	SNW domain-containing protein 1	Non-expressed	0.0383431	0.3842673	M300-M0
map04260	Cardiac muscle contraction	TR61414|c0_g1_i1|m.29124	K02267	cytochrome c oxidase subunit 6b	Non-expressed	0.0365945	0.3754118	M300-NM300
		TR141539|c0_g1_i1|m.98395	K00417	ubiquinol-cytochrome c reductase subunit 7	Non-expressed	0.0365945	0.3754118	M300-NM300
map05231	Choline metabolism in cancer	TR109938|c0_g1_i2|m.71087	K01115	phospholipase D1/2 [EC:3.1.4.4];	Newly-expressed	0.03025	0.3754118	M300-NM300
		TR138836|c1_g1_i2|m.95935	K00901	diacylglycerol kinase (ATP) [EC:2.7.1.107];	Newly-expressed	0.03025	0.3754118	M300-NM300

Four DAPs (1 upregulated protein, 1 newly expressed protein, 1 downregulated protein, and 1 non-expressed protein) were enriched in cysteine and methionine metabolism (map00270), among which TR79899|c1_g1_i1|m.44380 (aspartate aminotransferase, AAT) was upregulated, and TR105434|c2_g1_i2|m.66385 (methylthioribulose 1-phosphate dehydratase/enolase-phosphatase E1, DEP1) newly emerged. Four DAPs (1 upregulated protein, 2 downregulated proteins, and 1 non-expressed protein) were enriched in phenylalanine, tyrosine, and tryptophan biosynthesis (map00400), among which TR79899|c1_g1_i1|m.44380 (cytoplasmic, AAT) was upregulated. In taurine and hypotaurine metabolism (map00430), TR63112|c0_g1_i1|m.30616 (gamma-glutamyltranspeptidase/glutathione hydrolase/leukotriene-C4 hydrolase, GGT) newly emerged. In glycerophospholipid metabolism (map00564), four DAPs (3 newly expressed proteins and 1 non-expressed protein) were enriched, among which TR79915|c2_g1_i5|m.44422 (glycerol-3-phosphate O-acyltransferase 3/4, GPAT), TR109938|c0_g1_i2|m.71087 (phospholipase D1/2, PLD), and TR138836|c1_g1_i2|m.95935 (diacylglycerol kinase, DGK) were newly emerged proteins. In choline metabolism in cancer (map05231), TR109938|c0_g1_i2|m.71087(PLD) and TR138836|c1_g1_i2|m.95935 (DGK) were newly emerged proteins. Furthermore, 11 DAPs were enriched in the remaining four KEGG metabolic pathways, namely quorum sensing (map02024), biofilm formation – *P. aeruginosa* (map02025), spliceosome (map03040), and cardiac muscle contraction (map04260). However, all the 11 DAPs were either downregulated or non-expressed proteins.

To explore the function and relationship of the AM salt tolerance-related DAPs, including AAT, DEP1, GGT, GPAT, PLD and DGK, we constructed the protein–protein interaction network to elucidate the interaction of those target DAPs through String analysis. The results showed that there are 51 nodes and 285 edges in the protein–protein interaction network (PPI enrichment *p*-value: < 1.0e^-16^), as shown in **Figure 5**. Those target DAPs centrally concentrated in cysteine and methionine metabolism, glycerophospholipid metabolism, taurine and hypotaurine metabolism and phenylalanine, tyrosine and tryptophan biosynthesis in this network. Thus, the four pathways might play an important role in salt tolerance of mycorrhizal *E. angustifolia* seedlings.

## 4 Discussion

The resistance of plants to salt stress is a complex process, and its specific mechanism involves numerous internal factors in plants. Salt stress can affect the most important metabolic processes in plants, including photosynthesis, growth, energy and lipid metabolism, and protein synthesis. Plant responses to salt stress also require complex interactions of diverse genes, proteins, and metabolic or signaling pathways. In previous studies (Chang et al., 2018; Jia et al., 2019; Liang et al., 2021), the inoculation of AM fungi significantly affected many physiological indicators of *E. angustifolia* under salt stress, resulting in increased biomass of *E. angustifolia* seedlings, increased production of osmoregulatory substances, improved activity of antioxidant enzymes, decreased content of malondialdehyde, absorption and distribution of major mineral element ions, and enhanced photosynthesis. Therefore, AM fungi played an important role in improving the salt tolerance of *E. angustifolia*. Many studies have also reported the efficiency of AM fungi in improving the adaptability of host plants to salt stress and revealed the physiological and partial molecular mechanisms of AM fungi in enhancing nutrition and water absorption, plant growth hormone secretion, soluble substance accumulation, antioxidant enzyme activity, and photosynthesis in host plants (Evelin et al., 2009; Chen et al., 2017; Liang et al., 2021). However, the molecular mechanisms of AM symbiosis in alleviating salt stress in plants need to be further clarified.

Proteins are an essential component of organisms, serving as the material basis and direct functional executors of life activities. Proteomics research is helpful to better reveal a series of complex metabolic changes in AM symbiosis under salt stress and can provide new information for mining salt stress-responsive genes and comprehensively understanding the molecular mechanism of salt tolerance in AM plants. To elucidate the molecular mechanism of AM fungi in ameliorating the negative effects of salt stress, in this study, we conducted proteomic analysis of AM *E. angustifolia* under salt stress using an LFQ proteomics technique. A total of 170 DAPs related to salt tolerance in the roots of AM *E. angustifolia* seedlings were screened based on comparison between NM0 vs. NM300, NM0 vs. M0, M300 vs. M0, and M300 vs. NM300. Bioinformatics analysis showed that these DAPs were involved in reproduction, the cell membrane, the mitochondrion, the nucleus, the nucleoplasm, the cell wall, receptor binding, catalytic activity, transferase activity, and kinase activity. Furthermore, 20 of the 170 DAPs were mapped to nine KEGG metabolic pathways. Among them, the upregulated and newly expressed proteins were involved in five KEGG metabolic pathways related to amino acid metabolism, energy metabolism, and lipid metabolism.

### 4.1 DAPs related to amino acid metabolism

As the basic structural unit of proteins, amino acid is the precursor of the metabolites produced during plant growth and in response to various biotic and abiotic stresses. Our findings showed that the most important factors in the roots of AM *E. angustifolia* seedlings under salt stress were amino acid metabolism-related proteins. Among amino acid metabolism, cysteine and methionine metabolism (map00270) as well as phenylalanine, tyrosine, and tryptophan biosynthesis (map00400) increased significantly under salt stress. Moreover, 1 upregulated DAP (TR79899|c1_g1_i1|m.44380, AAT) and 1 newly expressed DAP (TR105434|c2_g1_i2|m.66385, methylthioribulose 1-phosphate dehydratase) were related to amino acid metabolism, as shown in **Table 3**.

**Figure 6:**
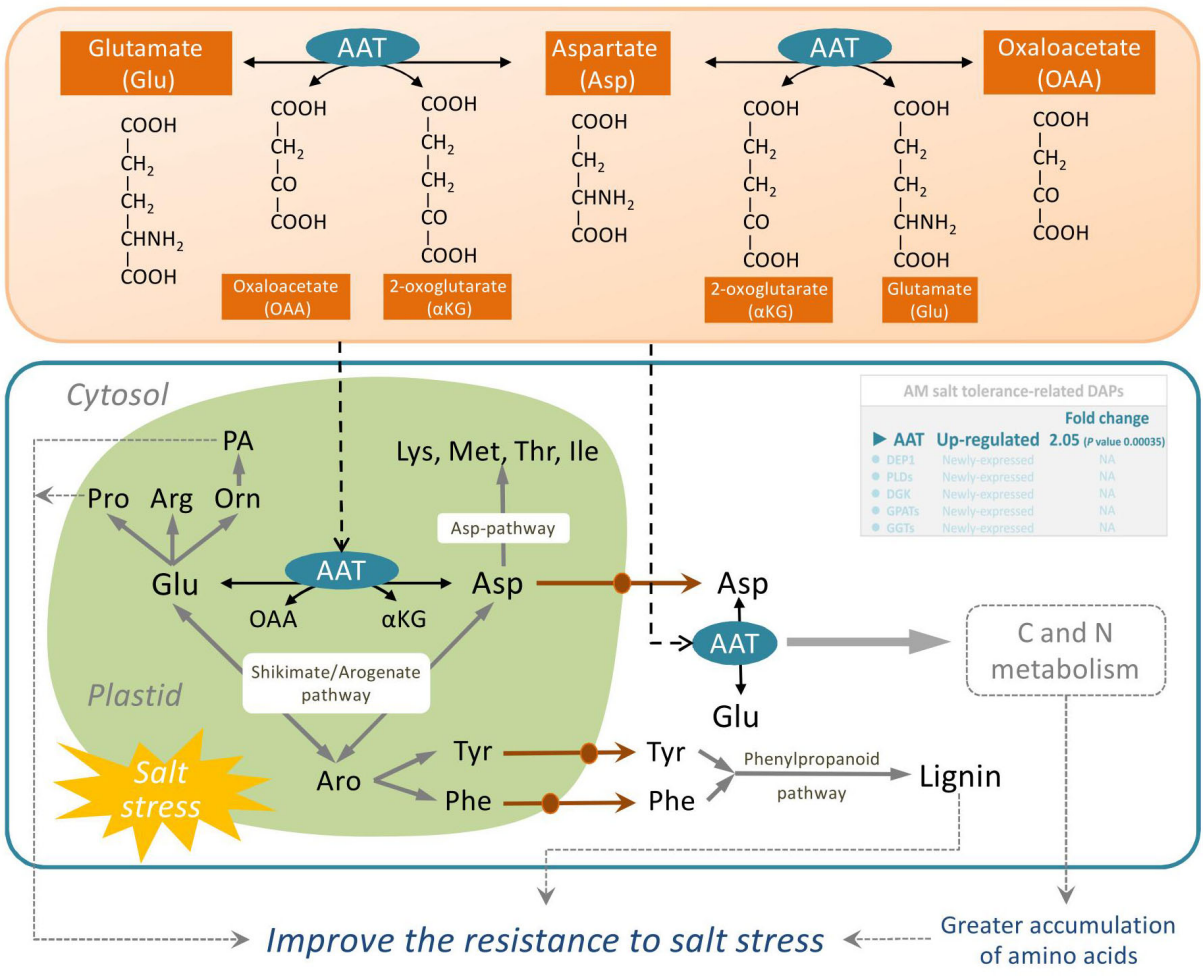
Schematic representation on the role of AAT in salt stress tolerance in mycorrhizal *E. angustifolia* seedlings. Abbreviations: AAT, aspartate aminotransferase; AS, asparagine synthetase; Glu, glutamate; Asp, aspartate; Lys, lysine; Met, methionine; Thr, threonine; Ile, isoleucine; Pro, proline; Arg, arginine; Orn, ornithine; Asn, asparagine; Tyr, tyrosine; Phe, phenylalanine; PA, polyamines; OAA, oxaloacetate; αKG, 2-oxoglutarate; Aro, arogenate.

AAT (EC: 2.6.1.1) widely exists in animals, plants, and microorganisms. It catalyses the reversible transamination between glutamate and oxaloacetate to generate aspartate and 2-oxoglutarate, and plays a key role in C and N metabolism of plants and the synthesis of amino acids from glutamate (de la Torre et al., 2014). Glutamate occupies a central position in amino acid metabolism in plants and is the substrate for the synthesis of glutamine from ammonia by glutamine synthetase. Furthermore, both the carbon skeleton and alpha-amino group of glutamate lay the foundation for the synthesis of gamma-aminobutyric acid, arginine, nucleotides, glutathione, chlorophyll, proline, and almost all nitrogenous compounds (Forde and Lea, 2007; Martínez-Andújar et al., 2013). Salt stress can affect the activities of nitrate reductase, glutamine synthetase, glutamate dehydrogenase, and glutamate synthase in plants (Ullah et al., 2019). Increased activity of AATs has been attributed to high glutamate demand and maintenance of the Krebs cycle to achieve the ideal C:N ratio under stress conditions (Naliwajski and Sklodowska, 2018). Some reports have suggested that salt stress enhances the activities of AAT enzymes in *Cucumis sativus*, *Jatropha curcas*, *Morus alba*, and *Glycine max* (Surabhi et al., 2008; Naliwajski and Sklodowska, 2018; Ullah et al., 2019). Our previous studies showed that the total proline and chlorophyll content in mycorrhizal *E. angustifolia* seedlings was significantly higher than that in non-mycorrhizal plants under salt stress, and AM fungi inoculation significantly improved the net photosynthesis rate of *E. angustifolia* seedlings (Liang et al., 2021). In this study, our data showed that salt stress induced the upregulation of AAT expression in mycorrhizal *E. angustifolia* seedlings, which may contribute to the greater accumulation of amino acids, polyamines, and lignin as well as the maintenance of ideal C:N ratio in mycorrhizal *E. angustifolia* seedlings under salt stress (**Figure 6**). These findings suggest that the accumulation levels of AAT proteins may be one of the reasons for increased salt tolerance in mycorrhizal *E. angustifolia* seedlings.

**Figure 7:**
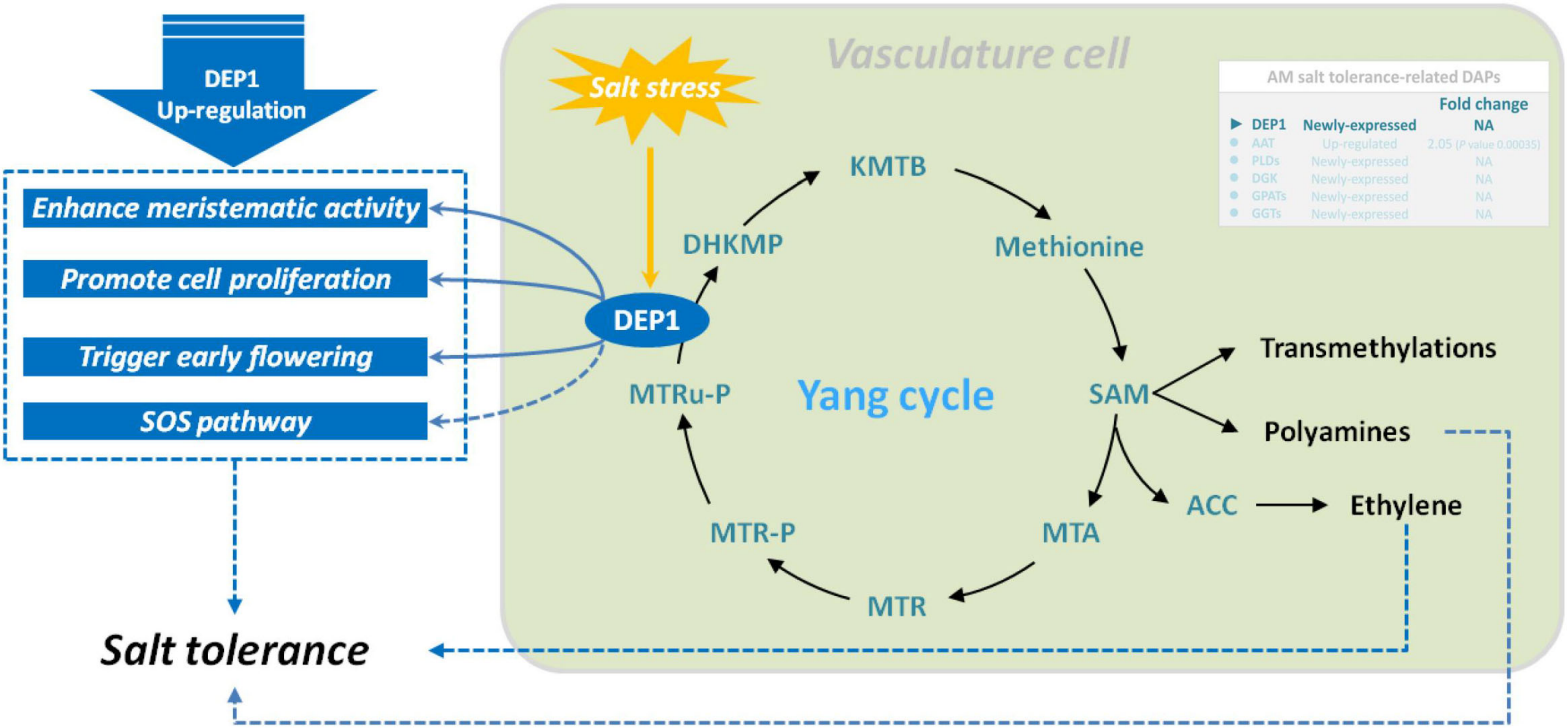
Schematic representation on the role of DEP1 in salt stress tolerance in mycorrhizal *E. angustifolia* seedlings. Abbreviations: DEP1, dehydratase-enolase-phosphatase 1; MTRu-P, 5’-methylthioribulose-1-phosphate; DHKMP, 1,2-dihidroxy-3-keto-5’-methylthiopentene; KMTB, 2-keto-4-methylthiobutyrate; MTR, 5’-methylthioribose; MTR-P, MTR-phosphate; MTA, 5’-methylthioadenosine; SAM, S-adenosyl-L-methionine; ACC, 1-aminocyclopropane-1-carboxylic acid.

Dehydratase-enolase-phosphatase 1 (DEP1, EC:4.2.1.109) is a trifunctional cytosolic enzyme localized in vascular and developing tissues. It possesses dehydratase, enolase, and phosphatase activities and directly catalyzes the conversion of 5-methylthioribulose-1-P (MTRu-1-P) to 1,2-dihydro-3-keto-5-methyl-thiopentene (DHKMP) without producing intermediate products in higher plants (Pommerrenig et al., 2011). As a plant-specific protein, DEP1, which is expressed preferentially in the vasculature, plays crucial roles in the Yang cycle, which is essential for not only ethylene biosynthesis but also polyamine and nicotianamine/phytosiderophore biosynthetic reactions in higher plants (Pattyn et al., 2020). Previous studies found that DEP1 is essential in the regulation of leaf senescence in apple (*Malus domestica*) (Hu et al., 2020) and in the biosynthesis of polyamines required for flowering and seed development in *Arabidopsis* (Zierer et al., 2015), enhances grain yield by increasing meristematic activity, and promotes cell proliferation in rice (Huang et al., 2009). Additionally, the expression of *DEP1*, a Yang cycle gene in apple, was positively induced by high salinity, and SOS pathway genes were remarkably upregulated in MdDEP1-overexpressing *Arabidopsis*, implying that DEP1 increases salt tolerance partly through the SOS pathway (Wang et al., 2019). Our previous studies found lower Na^+^ content and higher biomass accumulation in the root, stem, and leaves of mycorrhizal *E. angustifolia* seedlings compared with those in non-mycorrhizal seedlings under salt stress (Chang et al., 2018; He et al., 2020). In this study, our data showed that salt stress induced DEP1 expression in mycorrhizal *E. angustifolia* seedlings. Thus, we inferred that the newly expressed DEP1 could restrain the intake of Na^+^, maintain balanced K^+^/Na^+^ ratio, enhance meristematic activity, promote cell proliferation, and regulate polyamine and ethylene biosynthesis in mycorrhizal *E. angustifolia* seedlings under salt stress, as well as delay salt stress-induced damage to the root system of mycorrhizal *E. angustifolia* seedlings (**Figure 7**).

### 4.2 DAPs related to lipid metabolism

Lipids are important cell components that participate in various physiological processes in plants, such as signal transduction, skeleton rearrangement, and membrane transport, and play an important role in plant cell survival, growth, and stress responses. In this study, three mycorrhizal symbiotic DAPs related to salt tolerance in mycorrhizal *E. angustifolia* seedlings were shown to be involved in lipid metabolism. As shown in **Table 3**, three newly expressed DAPs (TR109938|c0_g1_i2|m.71087, phospholipase D; TR138836|c1_g1_i2|m.95935, diacylglycerol kinase (ATP); and TR79915|c2_g1_i5|m.44422, glycerol-3-phosphate O-acyltransferase) were enriched in the glycerolphospholipid metabolism pathway (map00564).

**Figure 8:**
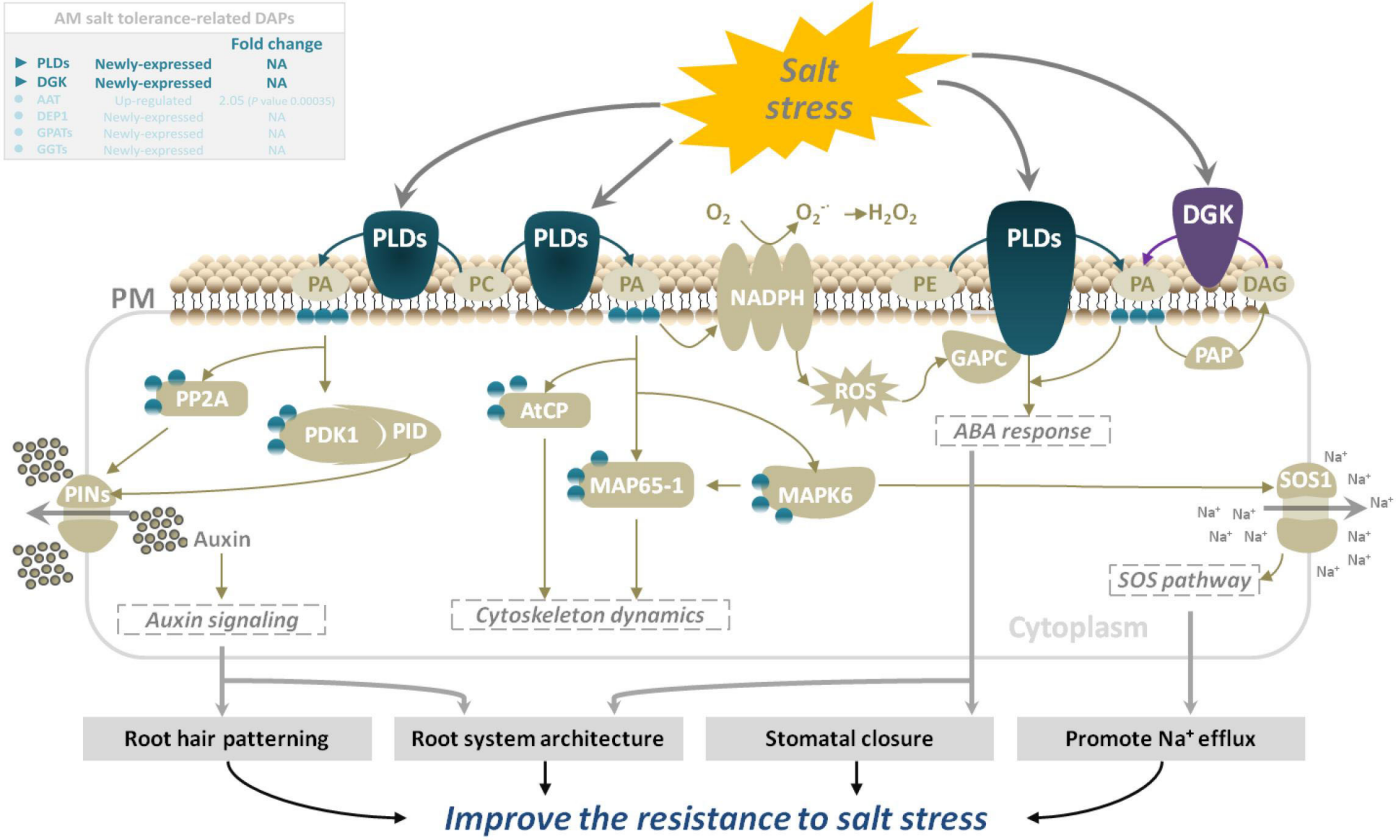
Schematic representation on the role of PLDs and DGK in salt stress tolerance in mycorrhizal *E. angustifolia* seedlings. Abbreviations: PLDs, Phospholipases D; DGK, diacylglycerol kinase; PM, plasma membrane; PA, phosphatidic acid; PC, phosphatidylcholine; PE, phosphatidylethanolamine; DAG, diacylglycerol; PAP, phosphatidic acid phosphatase;NADPH, NADPH oxidase; PP2A, protein phosphatase 2a; PDK1, phosphoinositide-dependent kinase 1; PID, PINOID protein kinase; PINs, pin-formed proteins; AtCP, arabidopsis actin capping protein; MAP65-1, 65-kDa microtubule-associated protein 1; MAPK6, mitogen-activated protein kinase 6; ROS, reactive oxygen species; GAPC, glyceraldehyde-3-phosphate dehydrogenase; SOS1, salt overly sensitive 1.

Phospholipase D (PLD, EC 3.1.4.4) is the most important type of phospholipase in plants. It has a wide range of substrates and can specifically catalyze the hydrolysis of the phosphate diester bond at the end of phospholipid molecules to generate phosphatidic acid (PA) and a head group. PA is believed to act as an important second messenger that converts and transmits extracellular signals to trigger cascade reactions during various stress responses (Bargmann et al., 2008). PLDs are widely involved in plant growth, development, and response to abiotic and biotic stresses, including stomatal closure, signal transduction, root elongation, salinity, cold and drought stress responses and other physiological processes, and plays an important role (Hong et al., 2016). PLDs have been reported to be involved in salt stress in several independent studies. Under salt stress, PLDs are induced to produce PA which binds to mitogen-activated protein kinase 6 (MPK6) and stimulates its kinase activity. MPK6 phosphorylates the SOS1 Na^+^/; H^+^ antiporter, and MPK6-SOS1 regulates Na^+^ exclusion from cells and stomatal closure to reduce the transpiratory steam of Na^+^ to leaves (Yu et al., 2010). In addition, PLDs regulate salt stress response by promoting root growth in *Arabidopsis* (Hong et al., 2008). DGK (EC: 2.7.1.107) is an important signaling kinase present in all higher plants. It can phosphorylate diacylglycerol to produce PA, which then participates in diverse biotic and abiotic stress responses (Escobar-Sepúlveda et al., 2017). In plant cells, diacylglycerol is a well-characterized component of the cell membrane that binds secondary messenger molecules in higher plants. In most cases, divalent cations such as Ca^2+^ can improve its enzyme activity. Our previous studies reported that mycorrhizal *E. angustifolia* seedlings maintained higher K^+^:Na^+^ ratios, Ca^2+^ concentration, and root dry weight than non-mycorrhizal seedlings under salt stress (Chang et al., 2018). In this study, salt stress induced the expression of PLDs and DGK in mycorrhizal *E. angustifolia* seedlings. The newly expressed PLDs and DGK are likely to play vital roles in enhancing salt tolerance in mycorrhizal *E. angustifolia* seedlings by regulating PA production, Na^+^ efflux, root growth, membrane structure stability, and the dynamic balance of lipid composition (**Figure 8**).

**Figure 9:**
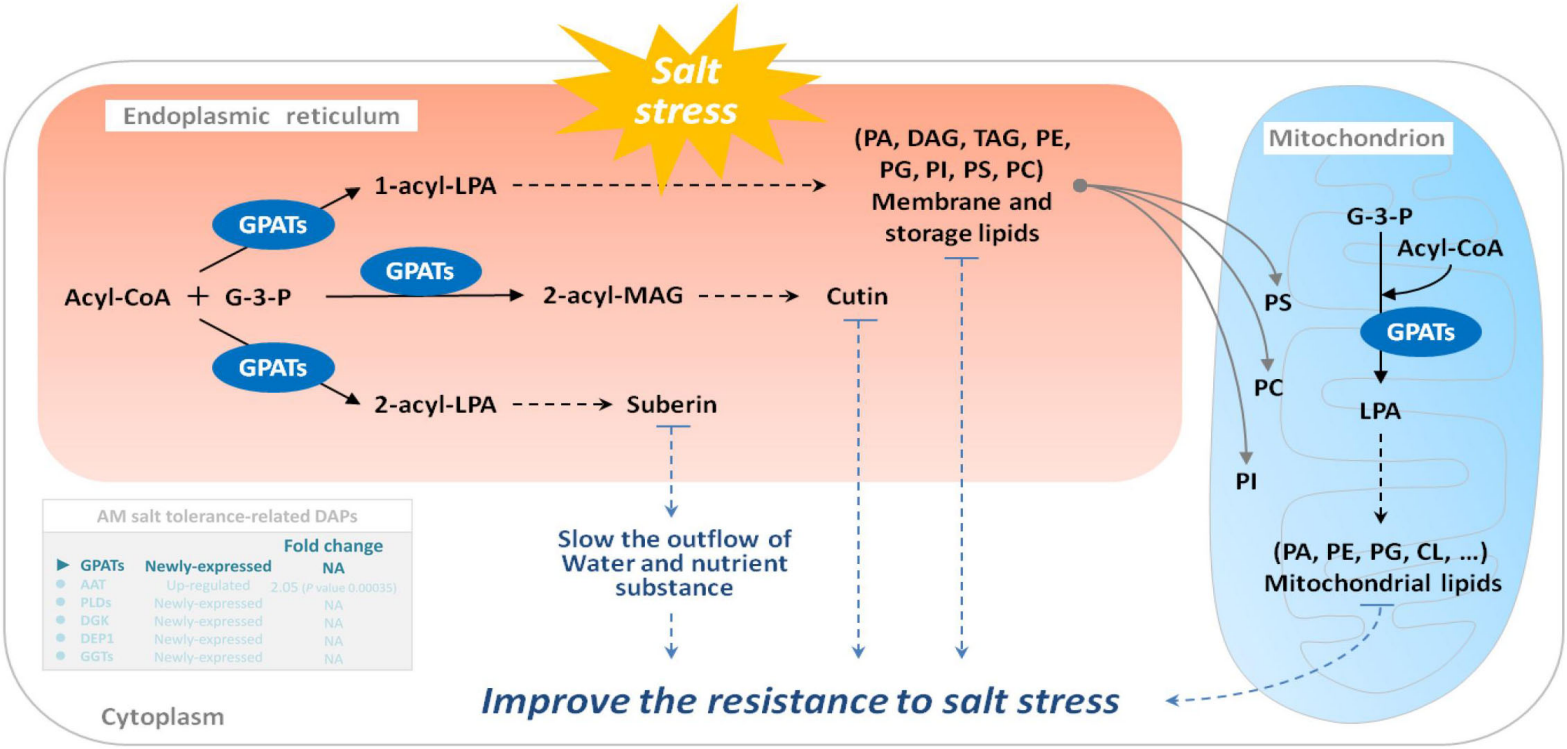
Schematic representation on the role of GPATs in salt stress tolerance in mycorrhizal *E. angustifolia* seedlings. Abbreviations: GPATs, glycerol-3-phosphate O-acyltransferases; G-3-P, glycerol-3-phosphate; Acyl-CoA, acyl-coenzyme A; LPA, lysophosphatidic acid; MAG, monoacylglycerol; PA, phosphatidic acid; DAG, diacylglycerol; TAG, triacylglycerol; PE, phosphatidylethanolamine; PG, phosphatidylglycerol; PI, phosphatidylinositol; PS, phosphatidylserine; PC, phosphatidylcholine; CL, cardiolipin.

Under salt stress, the first system that plants respond to is biofilm. Salt stress changes membrane fluidity, causes membrane peroxidation, destroys membrane structure, and finally leads to increased membrane permeability. Salt stress will change the relative content of fatty acid composition of the cell membrane, leading to a decrease in polyunsaturated fatty acid content with increasing salinity. GPAT (EC: 2.3.1.15) is a key enzyme in the synthesis of plant polyunsaturated fatty acids (PUFAs), which are important constituents of cell membrane lipids and play an important role in the resistance of plants to salt stress (Na et al., 2017). In plants, it catalyzes the formation of PA from saturated fatty acids and the synthesis of phosphatidylglycerol and other glycerols. An increase in GPAT activity increases the content of polyunsaturated fatty acids in plants. Research on salt tolerance of *Suaeda salsa*, tomato, and *Arabidopsis thaliana* (Sun et al., 2010; Na et al., 2017) showed that an increase in the content of unsaturated fatty acids can increase the salt tolerance of plants. Our previous studies also revealed that *E. angustifolia* seedlings inoculated with AM fungi had significantly lower malondialdehyde content (Liang et al., 2021), more organelles and intact cytoplasmic membranes, and no plasmic wall separation in root cells (He et al., 2020), and higher PSII activity than non-mycorrhizal seedlings under salt stress (Liang et al., 2021). In this study, the newly expressed GPAT may be helpful to increase the content of polyunsaturated fatty acids, thereby maintaining the membrane function, protecting the photosystem, maintaining a relatively intact cell structure, and ultimately alleviating the degree of membrane peroxidation in mycorrhizal *E. angustifolia* seedlings under salt stress (**Figure 9**).

### 4.3 DAPs related to energy metabolism

**Figure 10:**
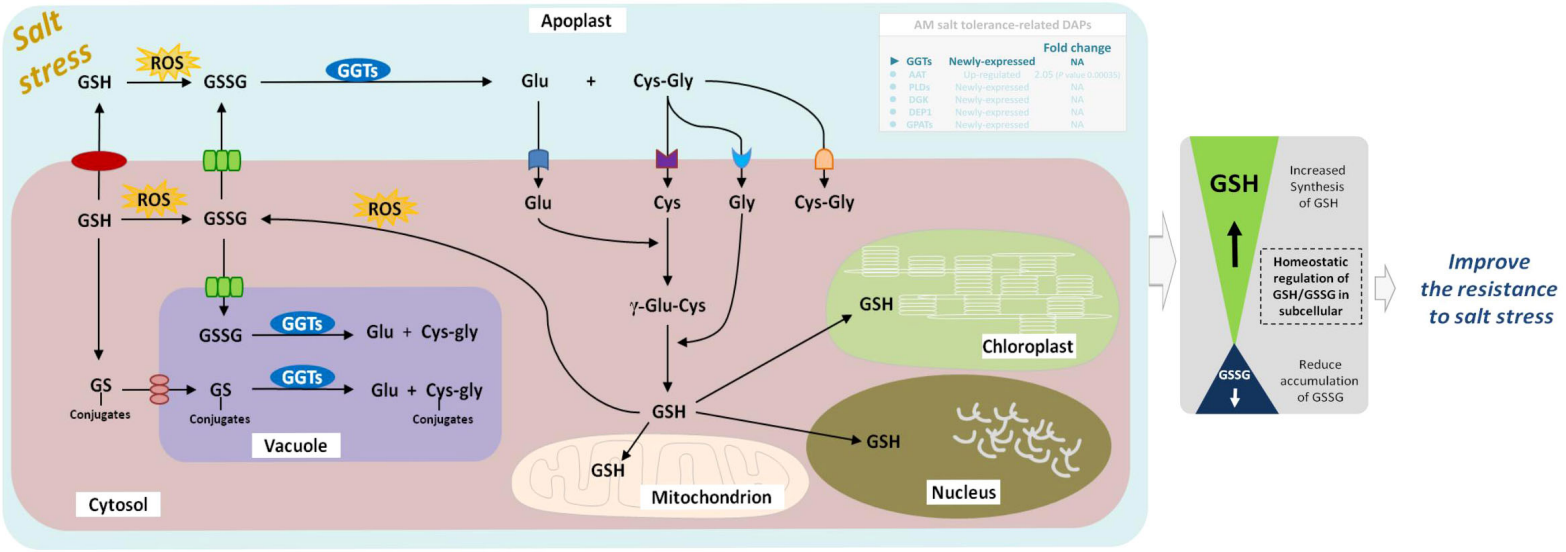
Schematic representation on the role of GGTs in salt stress tolerance in mycorrhizal *E. angustifolia* seedlings. Arrows indicate intracellular metabolic fluxes and intercompartment transport. Metabolites are in black, and enzymes in white. For simplicity, not all possible reactions are shown in the figure. GSH is synthesized in the cytosol by the enzymes GSH from Glu, Cys and Gly. Degradation of GSSG occurs in the apoplast and vacuole by GGTs. This pathway recycles Glu, Cys and Gly, which can be taken up by the cell and serve in GSH re-synthesis. GGTs catalyzes the degradation of GS conjugates in the vacuole. GSH pools are present in mitochondrion, chloroplast, apoplast, cytosol and nucleus. Abbreviations: GGTs, gamma-glutamyl transpeptidases; GSSG, glutathione disulphide; GSH, glutathione; GS-conjugates, glutathione S-conjugates; Glu, glutamate; Gly, glycine; Cys, cysteine; ROS, reactive oxygen species.

GGTs (EC:2.3.2.2) are part of the cell antioxidant defense mechanism. It promotes the apoplastic and vacuolar glutathione (GSH) degradation, provides cells with a local cysteine supply, and contributes to maintaining intracelular GSH levels. As a vital metabolite for various physiological processes in plants under abiotic stress, GSH is involved in the control of ROS with ascorbate and the detoxification of methylglyoxal, and is oxidized to GSSG (Hasanuzzaman et al., 2019). Several studies have shown that apoplastic GGTs may play an important role in countering oxidative stress or salvaging excreted GSSG (Destro et al., 2010), and vacuolar GGTs may be involved in the breakdown of glutamine synthetase-conjugates (Ohkama-Ohtsu et al., 2007). GGT1 and GGT2, isoforms of GGTs, are both localized in the apoplast, participate in the γ-glutamyl cycle, and degrade extracellular GSSG into Glu and Cys-Gly. The dipeptide Cys-Gly can be further hydrolyzed to Cys and Gly by an uncharacterized dipeptidase whose existence is inferred. The Glu, Cys, and Gly are then translocated to the cytosol as substrates for re-synthesis of GSH, followed by a novel round of export/degradation in the apoplast (Dorion et al., 2021). In the vacuole, GGT4 degrades the glutamine synthetase conjugates formed by glutathione S-transferase into Glu and Cys-Gly conjugates under herbicides stress (Grzam et al., 2007). Additionly, biochemical and quantitative proteomics studies in *Arabidopsis* showed that GGT1 also plays a role in redox signaling from the extracellular environment to internal compartments and transmits the redox information important for the adaptation of plants to their environment (Tolin et al., 2013). Recently, *GGT1* in *Arabidopsis* roots was shown to be induced by heavy metal stress, especialy during the first 24 h of stress (Deckers et al., 2020). At present, the gamma-glutamyl cycle remains poorly characterized in plants. GGTs are the only enzymes capable of degrading GSH in extra-cytosolic spaces (Giaretta et al., 2017); however, little is known about the role of GGTs in plant physiology under abiotic stress, including salt, drought, heat, cold, and heavy metal stresses. Our previous studies found that *E. angustifolia* seedlings inoculated with AM fungi had significantly lower malondialdehyde content than non-mycorrhizal seedlings under salt stress (Liang et al., 2021). In this study, GGTs were newly expressed in the roots of mycorrhizal *E. angustifolia* seedlings under salt stress. Under salt stress, GGTs might play an important role in mitigating oxidative stress by metabolizing GSSG and GS-conjugates and re-synthesizing GSH (**Figure 10**). In this context, it is possible that the newly expressed GGTs contribute to alleviating membrane peroxidation, thus lowering malondialdehyde content in mycorrhizal *E. angustifolia* seedlings under salt stress.

## 5 Conclusion

**Figure 11:**
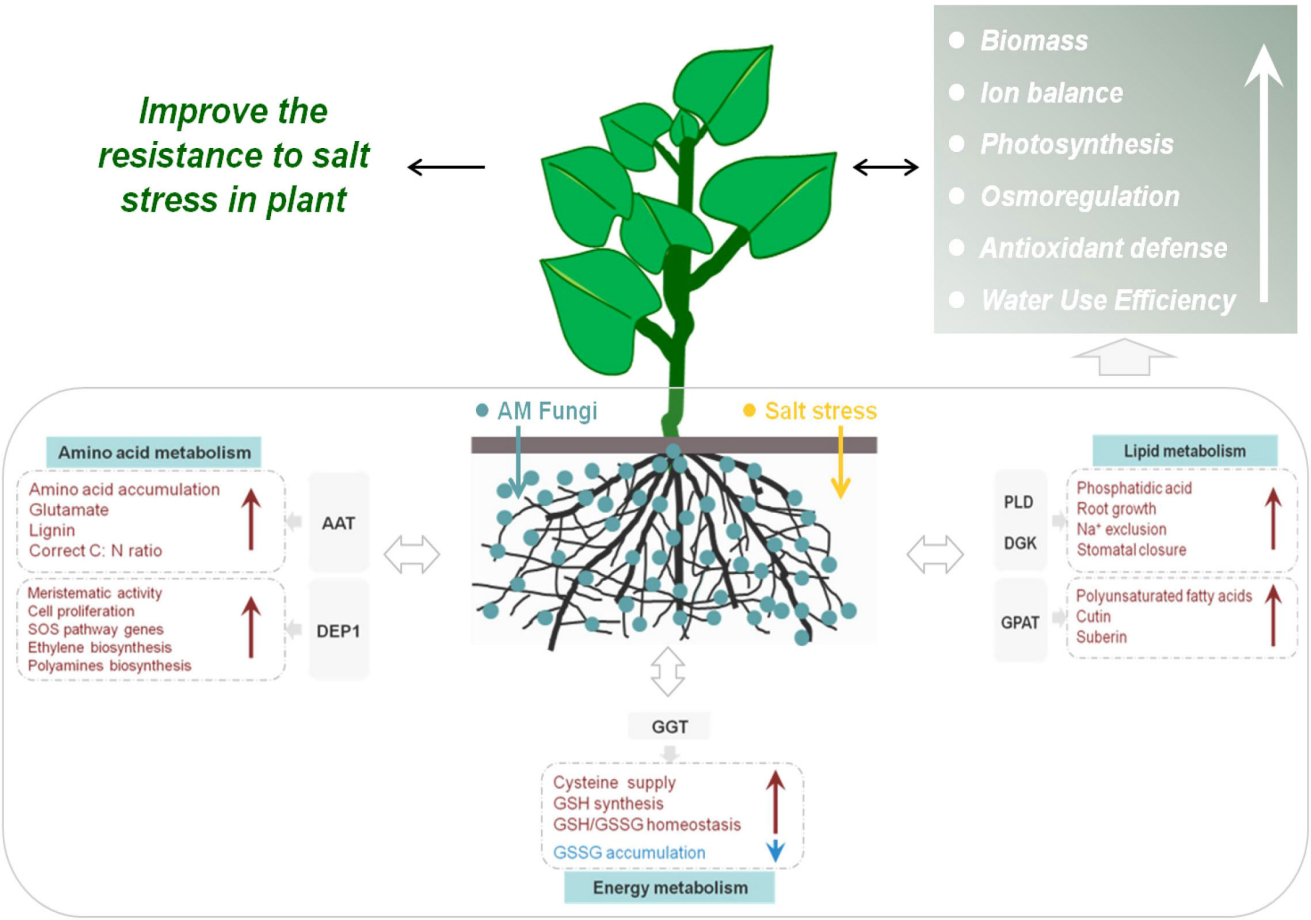
Schematic representation of salt-tolerance mechanisms in mycorrhizal *E. angustifolia* seedlings. Abbreviations: AAT, aspartate aminotransferase; GPAT, glycerol-3-phosphate O-acyltransferase; DEP1, Dehydratase-enolase-phosphatase 1; PLD, phospholipases D; DGK, diacylglycerol kinase; GGT, gamma-glutamyl transpeptidase; GSSG, glutathione disulphide; GSH, glutathione.

Our study investigated the molecular mechanisms of salt tolerance in mycorrhizal *E. angustifolia* seedlings under salt stress by proteomics analysis. LFQ proteomics provided an overview of the global proteomic responses of *E. angustifolia* roots to salt stress and AM fungi inoculation. Analysis of proteomic data revealed that 170 DAPs responded to AM fungi exclusively under salt stress, which offered molecular evidence of salt tolerance initiated by AM fungi. It seems that mycorrhizal symbiosis helps the host plant, *E. angustifolia*, to respond positively to salt stress and enhances its salt tolerance by regulating the activities of some key proteins related to amino acid metabolism, lipid metabolism, and glutathione metabolism in root tissues.As shown in **Figure 11**, AAT, DEP1, PLDs, DGKs, GPATs, and GGTs may play important roles in mitigating the detrimental effect of salt stress on mycorrhizal *E. angustifolia*. Our findings provide important insights into the salt tolerance mechanism induced by AM fungi and form the solid basis for future enhancement of plant tolerance to salinity stress.

## Data availability statement

The data presented in the study are deposited in the ProteomeXchange Consortium (http://proteomecentral.proteomexchange.org) via the iProX partner repository, accession number PXD038883.

## Author contributions

Experiments were designed by WC and FS; Statistical analysis were performed by WC. The manuscript was written by WC and YZ; and revised by YP, KL, DQ and FS. All authors contributed to the article and approved the submitted version.
