# Supplementary material for: Label-free quantitative proteomics of arbuscular mycorrhizal Elaeagnus angustifolia seedlings provides insights into salt-stress tolerance mechanisms
Source: Front Plant Sci. 2023 Jan 10;13:1098260. doi: 10.3389/fpls.2022.1098260 (PMC9873384; doi:10.3389/fpls.2022.1098260)
Supplement: Supplementary file 1 [file Table_1.docx]

# Suppl.Table 1︱Biofunction of Roots' Salt Tolerance-Related 170 Differentially Abundant Proteins (DAPs) in Mycorrhizal *E. angustifolia* Seedlings

| **Protein ID** | **Protein name and description** | **GO term** | **Category** | **GO ID** | **Differential expression** | ***P* value** | **Comparision group** |
| --- | --- | --- | --- | --- | --- | --- | --- |
| TR94455\|c0_g1_i1\|m.56869 | Integral to endoplasmic reticulum | membrane | Cellular component | GO:0016020 | Up-regulated | 0.0486499 | M300-M0 |
| TR92388\|c1_g1_i3\|m.54839 | transmembrane 205 | membrane | Cellular component | GO:0016020 | Up-regulated | 0.0486499 | M300-M0 |
| TR74481\|c0_g1_i2\|m.39771 | transmembrane DDB_G0292058 | membrane | Cellular component | GO:0016020 | Up-regulated | 0.0486499 | M300-M0 |
| TR7126\|c0_g1_i1\|m.3325 | CASP 1E2 | membrane | Cellular component | GO:0016020 | Up-regulated | 0.0486499 | M300-M0 |
| TR66091\|c0_g1_i1\|m.33163 | Late embryogenesis abundant (LEA) hydroxyproline-rich glyco | membrane | Cellular component | GO:0016020 | Up-regulated | 0.0486499 | M300-M0 |
| TR127518\|c0_g1_i1\|m.85891 | pyrophosphate-energized vacuolar membrane proton pump 1-like | membrane | Cellular component | GO:0016020 | Up-regulated | 0.0486499 | M300-M0 |
| TR103702\|c0_g1_i1\|m.64799 | BRASSINOSTEROID INSENSITIVE 1-associated receptor kinase 1 | membrane | Cellular component | GO:0016020 | Up-regulated | 0.0486499 | M300-M0 |
| TR9819\|c0_g1_i1\|m.4481 | phosphoglycerate cytosolic-like | nucleus | Cellular component | GO:0005634 | Up-regulated | 0.0401827 | M300-M0 |
| TR8332\|c0_g1_i1\|m.3844 | non-symbiotic hemoglobin 1 | nucleus | Cellular component | GO:0005634 | Up-regulated | 0.0401827 | M300-M0 |
| TR66550\|c0_g1_i1\|m.33529 | DNA-directed RNA polymerases IV and V subunit 9A | nucleoplasm | Cellular component | GO:0005654 | Up-regulated | 0.0239981 | M300-M0 |
| TR82728\|c0_g1_i3\|m.47193 | phosphoribosylamine--glycine ligase | catalytic activity | Molecular function | GO:0003824 | Up-regulated | 0.0293628 | M300-M0 |
| TR156744\|c0_g1_i1\|m.106231 | fructose-bisphosphate aldolase cytoplasmic isozyme | catalytic activity | Molecular function | GO:0003824 | Up-regulated | 0.0293628 | M300-M0 |
| TR109680\|c2_g1_i1\|m.70491 | codeine O-demethylase-like | catalytic activity | Molecular function | GO:0003824 | Up-regulated | 0.0293628 | M300-M0 |
| TR109247\|c0_g1_i1\|m.69768 | SNF1-related kinase regulatory subunit gamma-1 | catalytic activity | Molecular function | GO:0003824 | Up-regulated | 0.0293628 | M300-M0 |
| TR92676\|c0_g1_i2\|m.55081 | -L-isoaspartate O-methyltransferase 1-like | transferase activity | Molecular function | GO:0016740 | Up-regulated | 0.0272254 | M300-M0 |
| TR79899\|c1_g1_i1\|m.44380 | aspartate cytoplasmic | transferase activity | Molecular function | GO:0016740 | Up-regulated | 0.0272254 | M300-M0 |
| TR138382\|c1_g1_i1\|m.95586 | granule-bound starch synthase chloroplastic amyloplastic-like | transferase activity | Molecular function | GO:0016740 | Up-regulated | 0.0272254 | M300-M0 |
| TR124267\|c1_g4_i2\|m.82959 | G-type lectin S-receptor-like serine threonine- kinase At4g27290 isoform X1 | kinase activity | Molecular function | GO:0016301 | Up-regulated | 0.0486499 | M300-NM300 |
| TR131581\|c1_g1_i1\|m.89750 | aspartyl protease AED3-like | cell wall | Cellular component | GO:0005618 | Up-regulated | 0.0486499 | M300-NM300 |
| TR101711\|c0_g1_i2\|m.62927 | non-classical arabinogalactan 30 | cell wall | Cellular component | GO:0005618 | Up-regulated | 0.0486499 | M300-NM300 |
| TR138997\|c4_g1_i2\|m.96070 | CASP 1D1 | membrane | Cellular component | GO:0016020 | Up-regulated | 0.0486499 | M300-NM300 |
| TR56157\|c0_g1_i1\|m.25926 | peroxidase 47 | membrane | Cellular component | GO:0016020 | Up-regulated | 0.0486499 | M300-NM300 |
| TR97371\|c0_g1_i2\|m.58221 | geraniol 8-hydroxylase-like | membrane | Cellular component | GO:0016020 | Down-regulated | 0.0486499 | M300-M0 |
| TR9561\|c0_g1_i2\|m.4378 | reticulon B2-like isoform 2 | membrane | Cellular component | GO:0016020 | Down-regulated | 0.0486499 | M300-M0 |
| TR91562\|c0_g1_i1\|m.53942 | flavonoid 3 ,5 -hydroxylase | membrane | Cellular component | GO:0016020 | Down-regulated | 0.0486499 | M300-M0 |
| TR68724\|c2_g2_i1\|m.35201 | reticulon B1-like | membrane | Cellular component | GO:0016020 | Down-regulated | 0.0486499 | M300-M0 |
| TR20634\|c0_g1_i1\|m.9280 | ABC transporter A family member 2 | membrane | Cellular component | GO:0016020 | Down-regulated | 0.0486499 | M300-M0 |
| TR158804\|c0_g1_i1\|m.106898 | F-ATPase beta partial (mitochondrion) | membrane | Cellular component | GO:0016020 | Down-regulated | 0.0401827 | M300-M0 |
| TR141458\|c0_g1_i1\|m.98312 | altered inheritance of mitochondria 32 | membrane | Cellular component | GO:0016020 | Down-regulated | 0.0401827 | M300-M0 |
| TR131324\|c2_g1_i1\|m.89435 | glucose transporter | membrane | Cellular component | GO:0016020 | Down-regulated | 0.0043405 | M300-M0 |
| TR118526\|c2_g1_i1\|m.78078 | Sodium calcium exchanger family calcium-binding EF hand family isoform 1 | membrane | Cellular component | GO:0016020 | Down-regulated | 0.0239981 | M300-M0 |
| TR102444\|c0_g1_i1\|m.63987 | cytochrome b5 | membrane | Cellular component | GO:0016020 | Down-regulated | 0.0239981 | M300-M0 |
| TR92675\|c0_g1_i2\|m.55079 | splicing factor 3B subunit 3-like | nucleus | Cellular component | GO:0005634 | Down-regulated | 0.0239981 | M300-M0 |
| TR132658\|c2_g2_i2\|m.91794 | WEB family chloroplastic-like | nucleus | Cellular component | GO:0005634 | Down-regulated | 0.0293628 | M300-M0 |
|  |  | reproduction | Biological process | GO:0000003 | Down-regulated | 0.04287081 | M300-NM300 |
| TR24767\|c0_g1_i1\|m.11347 | tubulin beta chain isoform X2 | receptor binding | Molecular function | GO:0005102 | Down-regulated | 0.0293628 | M300-M0 |
| TR93544\|c2_g1_i4\|m.56509 | rRNA 2 -O-methyltransferase fibrillarin 2-like isoform 1 | nucleoplasm | Cellular component | GO:0005654 | Down-regulated | 0.0293628 | M300-M0 |
| TR69588\|c0_g1_i1\|m.36222 | serine arginine-rich splicing factor SR30-like isoform X2 | nucleoplasm | Cellular component | GO:0005654 | Down-regulated | 0.0293628 | M300-M0 |
| TR108357\|c0_g1_i1\|m.68619 | heterogeneous nuclear ribonucleo R-like | nucleoplasm | Cellular component | GO:0005654 | Down-regulated | 0.0293628 | M300-M0 |
| TR93551\|c0_g1_i1\|m.56528 | 4-hydroxy-3-methylbut-2-enyl diphosphate synthase isoform 1 | catalytic activity | Molecular function | GO:0003824 | Down-regulated | 0.0293628 | M300-M0 |
| TR86243\|c0_g1_i1\|m.49606 | 12-oxophytodienoate reductase 2-like | catalytic activity | Molecular function | GO:0003824 | Down-regulated | 0.0293628 | M300-M0 |
| TR79213\|c0_g2_i4\|m.43558 | thiamine thiazole chloroplastic-like | catalytic activity | Molecular function | GO:0003824 | Down-regulated | 0.0293628 | M300-M0 |
| TR54215\|c0_g1_i2\|m.24930 | phenylalanine ammonia lyase | catalytic activity | Molecular function | GO:0003824 | Down-regulated | 0.0293628 | M300-M0 |
| TR143653\|c0_g1_i1\|m.101059 | 12-oxophytodienoate reductase 11 | catalytic activity | Molecular function | GO:0003824 | Down-regulated | 0.0272254 | M300-M0 |
| TR143362\|c0_g1_i3\|m.100535 | anthranilate synthase component I- chloroplastic-like | catalytic activity | Molecular function | GO:0003824 | Down-regulated | 0.0272254 | M300-M0 |
| TR135538\|c0_g1_i2\|m.93124 | long chain acyl- synthetase chloroplastic | catalytic activity | Molecular function | GO:0003824 | Down-regulated | 0.0486499 | M300-M0 |
| TR130044\|c0_g1_i1\|m.88155 | tryptophan decarboxylase | catalytic activity | Molecular function | GO:0003824 | Down-regulated | 0.0486499 | M300-M0 |
| TR109132\|c1_g1_i1\|m.69562 | phenylalanine ammonia-lyase | catalytic activity | Molecular function | GO:0003824 | Down-regulated | 0.0486499 | M300-M0 |
| TR79846\|c2_g2_i5\|m.44255 | S-adenosylmethionine synthase 2 | transferase activity | Molecular function | GO:0016740 | Down-regulated | 0.0486499 | M300-M0 |
| TR132556\|c2_g1_i1\|m.91459 | 2-dehydro-3-deoxyphosphoheptonate aldolase 3-deoxy-d-arabino-heptulosonate 7-phosphate synthetase | transferase activity | Molecular function | GO:0016740 | Down-regulated | 0.0486499 | M300-M0 |
| TR1610\|c0_g1_i1\|m.849 | mitochondrial pyruvate carrier 4 | mitochondrion | Cellular component | GO:0005739 | Down-regulated | 0.0486499 | M300-NM300 |
| TR103715\|c0_g1_i1\|m.64818 | mitochondrial import inner membrane translocase subunit TIM14-1 | mitochondrion | Cellular component | GO:0005739 | Down-regulated | 0.0486499 | M300-NM300 |
| TR88268\|c0_g1_i3\|m.52073 | chloroplastic-like isoform X1 | membrane | Cellular component | GO:0016020 | Newly-expressed | 0.0486499 | M300-M0 |
| TR79895\|c0_g1_i2\|m.44370 | ALA-interacting subunit 3-like | membrane | Cellular component | GO:0016020 | Newly-expressed | 0.0486499 | M300-M0 |
| TR79682\|c1_g1_i2\|m.44069 | vam6 Vps39 -like | membrane | Cellular component | GO:0016020 | Newly-expressed | 0.0486499 | M300-M0 |
| TR69091\|c6_g2_i1\|m.35624 | Mediator of RNA polymerase II transcription subunit 11 | membrane | Cellular component | GO:0016020 | Newly-expressed | 0.0486499 | M300-M0 |
| TR66547\|c1_g1_i2\|m.33526 | Ca2+ and calmodulin-dependent kinase | membrane | Cellular component | GO:0016020 | Newly-expressed | 0.0486499 | M300-M0 |
| TR63986\|c0_g1_i2\|m.31242 | cytochrome b5 | membrane | Cellular component | GO:0016020 | Newly-expressed | 0.0486499 | M300-M0 |
| TR63632\|c0_g1_i2\|m.30978 | TBC1 domain family member 17-like isoform X1 | membrane | Cellular component | GO:0016020 | Newly-expressed | 0.0486499 | M300-M0 |
| TR63112\|c0_g1_i1\|m.30616 | gamma-glutamyltranspeptidase 1 | membrane | Cellular component | GO:0016020 | Newly-expressed | 0.0486499 | M300-M0 |
| TR51957\|c0_g1_i1\|m.23950 | disulfide isomerase-like 1-4 | membrane | Cellular component | GO:0016020 | Newly-expressed | 0.0486499 | M300-M0 |
| TR44159\|c0_g1_i1\|m.20284 | membrin-11-like | membrane | Cellular component | GO:0016020 | Newly-expressed | 0.0486499 | M300-M0 |
| TR20416\|c0_g2_i1\|m.9195 | fasciclin-like arabinogalactan 11-like | membrane | Cellular component | GO:0016020 | Newly-expressed | 0.0486499 | M300-M0 |
| TR20194\|c0_g1_i1\|m.9110 | vacuolar sorting-associated 55 homolog | membrane | Cellular component | GO:0016020 | Newly-expressed | 0.0486499 | M300-M0 |
| TR165977\|c0_g1_i1\|m.109226 | aldo keto reductase | membrane | Cellular component | GO:0016020 | Newly-expressed | 0.0486499 | M300-M0 |
| TR143546\|c1_g1_i4\|m.100828 | SEC12 2 | membrane | Cellular component | GO:0016020 | Newly-expressed | 0.0486499 | M300-M0 |
| TR143305\|c0_g1_i1\|m.100426 | Peroxisomal membrane 22 kDa family isoform 2 | membrane | Cellular component | GO:0016020 | Newly-expressed | 0.0486499 | M300-M0 |
| TR143115\|c0_g2_i4\|m.100180 | Sterol methyltransferase 1 | membrane | Cellular component | GO:0016020 | Newly-expressed | 0.0486499 | M300-M0 |
|  |  | reproduction | Biological process | GO:0000003 | Newly-expressed | 0.04287081 | M300-NM300 |
| TR141166\|c0_g1_i1\|m.98076 | mitochondrial import inner membrane translocase subunit TIM23-2-like | membrane | Cellular component | GO:0016020 | Newly-expressed | 0.0486499 | M300-M0 |
| TR140080\|c1_g1_i1\|m.97211 | endoplasmic reticulum-Golgi intermediate compartment 3-like | membrane | Cellular component | GO:0016020 | Newly-expressed | 0.0486499 | M300-M0 |
| TR135724\|c1_g1_i1\|m.93518 | STRUBBELIG-RECEPTOR FAMILY 6-like | membrane | Cellular component | GO:0016020 | Newly-expressed | 0.0486499 | M300-M0 |
| TR132233\|c0_g1_i3\|m.90684 | lysine histidine transporter-like 8-like | membrane | Cellular component | GO:0016020 | Newly-expressed | 0.0486499 | M300-M0 |
| TR128649\|c0_g2_i2\|m.87055 | disulfide-isomerase 5-2 | membrane | Cellular component | GO:0016020 | Newly-expressed | 0.0486499 | M300-M0 |
| TR127349\|c0_g1_i2\|m.85756 | FATTY ACID EXPORT chloroplastic-like | membrane | Cellular component | GO:0016020 | Newly-expressed | 0.0486499 | M300-M0 |
| TR123584\|c0_g1_i2\|m.82623 | beta-glucosidase-like chloroplastic | membrane | Cellular component | GO:0016020 | Newly-expressed | 0.0486499 | M300-M0 |
| TR122506\|c0_g1_i1\|m.81477 | Uncharacterized protein TCM_005902 | membrane | Cellular component | GO:0016020 | Newly-expressed | 0.0401827 | M300-M0 |
| TR120440\|c0_g1_i1\|m.79739 | non-specific lipid-transfer | membrane | Cellular component | GO:0016020 | Newly-expressed | 0.0293628 | M300-M0 |
| TR118450\|c0_g1_i1\|m.77956 | Uro-adherence factor A | membrane | Cellular component | GO:0016020 | Newly-expressed | 0.0293628 | M300-M0 |
| TR114562\|c0_g1_i2\|m.74605 | aminoacylase-1 isoform X1 | membrane | Cellular component | GO:0016020 | Newly-expressed | 0.0293628 | M300-M0 |
| TR114501\|c0_g1_i2\|m.74524 | thiosulfate sulfurtransferase 18-like | membrane | Cellular component | GO:0016020 | Newly-expressed | 0.0293628 | M300-M0 |
| TR104013\|c0_g1_i1\|m.65097 | psbP domain-containing chloroplastic-like isoform X1 | membrane | Cellular component | GO:0016020 | Newly-expressed | 0.0293628 | M300-M0 |
| TR101337\|c3_g1_i1\|m.62060 | NADH dehydrogenase subunit 3 | membrane | Cellular component | GO:0016020 | Newly-expressed | 0.0293628 | M300-M0 |
| TR123452\|c0_g1_i1\|m.82426 | Serine-type peptidase | nucleus | Cellular component | GO:0005634 | Newly-expressed | 0.0293628 | M300-M0 |
| TR88426\|c0_g1_i2\|m.52472 | Aldo keto reductase | catalytic activity | Molecular function | GO:0003824 | Newly-expressed | 0.0293628 | M300-M0 |
| TR87244\|c0_g1_i2\|m.50534 | Tonoplast monosaccharide transporter2 isoform 1 | catalytic activity | Molecular function | GO:0003824 | Newly-expressed | 0.0293628 | M300-M0 |
| TR82423\|c0_g3_i9\|m.46701 | PREDICTED: uncharacterized protein YMR315W isoform X2 | catalytic activity | Molecular function | GO:0003824 | Newly-expressed | 0.0293628 | M300-M0 |
| TR81764\|c0_g1_i2\|m.45869 | L-idonate 5-dehydrogenase | catalytic activity | Molecular function | GO:0003824 | Newly-expressed | 0.0293628 | M300-M0 |
| TR7557\|c0_g1_i1\|m.3510 | 4-hydroxy-3-methylbut-2-enyl diphosphate reductase-like | catalytic activity | Molecular function | GO:0003824 | Newly-expressed | 0.0293628 | M300-M0 |
| TR74953\|c0_g1_i1\|m.39995 | monocopper oxidase SKU5-like | catalytic activity | Molecular function | GO:0003824 | Newly-expressed | 0.0272254 | M300-M0 |
| TR22423\|c0_g1_i1\|m.10187 | long chain acyl- synthetase 4-like | catalytic activity | Molecular function | GO:0003824 | Newly-expressed | 0.0272254 | M300-M0 |
| TR143909\|c1_g1_i1\|m.101843 | acyl-activating enzyme 15 | catalytic activity | Molecular function | GO:0003824 | Newly-expressed | 0.0272254 | M300-M0 |
| TR143422\|c1_g1_i3\|m.100668 | external alternative NAD(P)H-ubiquinone oxidoreductase mitochondrial | catalytic activity | Molecular function | GO:0003824 | Newly-expressed | 0.0272254 | M300-M0 |
| TR139794\|c2_g5_i1\|m.96821 | biotin carboxyl carrier of acetyl- chloroplastic-like | catalytic activity | Molecular function | GO:0003824 | Newly-expressed | 0.0272254 | M300-M0 |
| TR123556\|c0_g1_i1\|m.82567 | fruit pKIWI502-like | catalytic activity | Molecular function | GO:0003824 | Newly-expressed | 0.0272254 | M300-M0 |
| TR121236\|c0_g3_i1\|m.80366 | 12-oxophytodienoate reductase 2 | catalytic activity | Molecular function | GO:0003824 | Newly-expressed | 0.0272254 | M300-M0 |
| TR93508\|c0_g1_i1\|m.56404 | UDP-glycosyltransferase 74F2-like | transferase activity | Molecular function | GO:0016740 | Newly-expressed | 0.0486499 | M300-M0 |
| TR79915\|c2_g1_i5\|m.44422 | glycerol-3-phosphate acyltransferase 3-like | transferase activity | Molecular function | GO:0016740 | Newly-expressed | 0.0486499 | M300-M0 |
| TR79210\|c0_g1_i1\|m.43553 | HXXXD-type acyl-transferase family | transferase activity | Molecular function | GO:0016740 | Newly-expressed | 0.0486499 | M300-M0 |
| TR142628\|c2_g1_i2\|m.99535 | adenine phosphoribosyltransferase 2-like | transferase activity | Molecular function | GO:0016740 | Newly-expressed | 0.0486499 | M300-M0 |
| TR132735\|c1_g1_i7\|m.92118 | Fatty acid biosynthesis 1 isoform 1 | transferase activity | Molecular function | GO:0016740 | Newly-expressed | 0.0486499 | M300-M0 |
| TR122833\|c2_g2_i1\|m.81827 | Ankyrin repeat domain-containing 2 | transferase activity | Molecular function | GO:0016740 | Newly-expressed | 0.0486499 | M300-M0 |
| TR101579\|c0_g1_i3\|m.62531 | mannose-1-phosphate guanyltransferase alpha-like | transferase activity | Molecular function | GO:0016740 | Newly-expressed | 0.0486499 | M300-M0 |
| TR87266\|c0_g1_i1\|m.50560 | Receptor kinase FERONIA | kinase activity | Molecular function | GO:0016301 | Newly-expressed | 0.0486499 | M300-NM300 |
| TR47093\|c0_g1_i1\|m.21514 | vacuolar fusion CCZ1 homolog isoform X2 | kinase activity | Molecular function | GO:0016301 | Newly-expressed | 0.0486499 | M300-NM300 |
| TR138836\|c1_g1_i2\|m.95935 | diacylglycerol kinase 5-like | kinase activity | Molecular function | GO:0016301 | Newly-expressed | 0.0486499 | M300-NM300 |
| TR123157\|c0_g2_i1\|m.82102 | Nodulation receptor kinase | kinase activity | Molecular function | GO:0016301 | Newly-expressed | 0.0486499 | M300-NM300 |
| TR104651\|c0_g1_i2\|m.65608 | Alpha-glucan water chloroplastic | kinase activity | Molecular function | GO:0016301 | Newly-expressed | 0.0486499 | M300-NM300 |
| TR103312\|c0_g1_i1\|m.64552 | pfkB-type carbohydrate kinase family | kinase activity | Molecular function | GO:0016301 | Newly-expressed | 0.0486499 | M300-NM300 |
| TR139179\|c0_g1_i1\|m.96252 | probable inactive receptor kinase At1g48480 | kinase activity | Molecular function | GO:0016301 | Newly-expressed | 0.0486499 | M300-NM300 |
| TR62303\|c0_g1_i5\|m.29773 | ketol-acid chloroplastic-like | mitochondrion | Cellular component | GO:0005739 | Newly-expressed | 0.0486499 | M300-NM300 |
| TR143603\|c1_g3_i1\|m.100941 | Mitochondrial ribosomal L51 S25 CI-B8 family | mitochondrion | Cellular component | GO:0005739 | Newly-expressed | 0.0486499 | M300-NM300 |
| TR103261\|c0_g1_i3\|m.64488 | GDSL esterase lipase At5g14450-like | cell wall | Cellular component | GO:0005618 | Newly-expressed | 0.0486499 | M300-NM300 |
| TR119398\|c0_g1_i2\|m.78859 | Alpha-expansin 13 | cell wall | Cellular component | GO:0005618 | Newly-expressed | 0.0486499 | M300-NM300 |
| TR115234\|c1_g2_i5\|m.75634 | subtilisin-like protease isoform X1 | cell wall | Cellular component | GO:0005618 | Newly-expressed | 0.0486499 | M300-NM300 |
| TR87992\|c0_g1_i1\|m.51524 | subtilisin-like protease | membrane | Cellular component | GO:0016020 | Newly-expressed | 0.0486499 | M300-NM300 |
| TR69659\|c2_g1_i1\|m.36409 | signal peptide peptidase-like | membrane | Cellular component | GO:0016020 | Newly-expressed | 0.0486499 | M300-NM300 |
| TR58811\|c0_g2_i1\|m.27408 | Syntaxin 61 family | membrane | Cellular component | GO:0016020 | Newly-expressed | 0.0401827 | M300-NM300 |
| TR140607\|c0_g1_i1\|m.97879 | nucleotide binding | membrane | Cellular component | GO:0016020 | Newly-expressed | 0.0401827 | M300-NM300 |
| TR109938\|c0_g1_i2\|m.71087 | phospholipase D beta 1-like | membrane | Cellular component | GO:0016020 | Newly-expressed | 0.0401827 | M300-NM300 |
| TR109876\|c0_g2_i2\|m.70924 | UDP-glucuronic acid decarboxylase 1-like | membrane | Cellular component | GO:0016020 | Newly-expressed | 0.0401827 | M300-NM300 |
| TR93538\|c2_g1_i6\|m.56486 | probable sugar phosphate phosphate translocator At5g25400-like | membrane | Cellular component | GO:0016020 | Newly-expressed | 0.0401827 | M300-NM300 |
| TR98703\|c0_g1_i1\|m.59840 | N-terminal nucleophile aminohydrolase | membrane | Cellular component | GO:0016020 | Non-expressed | 0.0401827 | M300-M0 |
| TR92896\|c0_g2_i4\|m.55399 | NRT1 PTR FAMILY -like | membrane | Cellular component | GO:0016020 | Non-expressed | 0.0293628 | M300-M0 |
| TR80511\|c0_g1_i1\|m.44696 | hypothetical protein PRUPE_ppa011920mg | membrane | Cellular component | GO:0016020 | Non-expressed | 0.0293628 | M300-M0 |
| TR80192\|c1_g1_i1\|m.44518 | chlorophyll a-b binding chloroplastic | membrane | Cellular component | GO:0016020 | Non-expressed | 0.0293628 | M300-M0 |
| TR77976\|c1_g2_i2\|m.42531 | probable aquaporin PIP2-5 | membrane | Cellular component | GO:0016020 | Non-expressed | 0.0293628 | M300-M0 |
| TR75266\|c8_g1_i4\|m.40687 | atp synthase subunit mitochondrial | membrane | Cellular component | GO:0016020 | Non-expressed | 0.0293628 | M300-M0 |
| TR74524\|c0_g1_i1\|m.39857 | AAA-ATPase mitochondrial-like | membrane | Cellular component | GO:0016020 | Non-expressed | 0.0272254 | M300-M0 |
| TR74308\|c0_g1_i1\|m.39580 | cultured cell | membrane | Cellular component | GO:0016020 | Non-expressed | 0.0272254 | M300-M0 |
| TR69371\|c0_g1_i1\|m.36012 | SEC14 cytosolic factor family phosphoglyceride transfer family | membrane | Cellular component | GO:0016020 | Non-expressed | 0.0272254 | M300-M0 |
| TR66032\|c4_g4_i1\|m.33107 | probable methyltransferase PMT2-like | membrane | Cellular component | GO:0016020 | Non-expressed | 0.0486499 | M300-M0 |
| TR60150\|c1_g1_i1\|m.28346 | SEC14 cytosolic factor family phosphoglyceride transfer family | membrane | Cellular component | GO:0016020 | Non-expressed | 0.0486499 | M300-M0 |
| TR58688\|c1_g1_i2\|m.27318 | transportin-3-like isoform 1 | membrane | Cellular component | GO:0016020 | Non-expressed | 0.0486499 | M300-M0 |
| TR35218\|c0_g1_i1\|m.16135 | TT12-2 MATE transporter | membrane | Cellular component | GO:0016020 | Non-expressed | 0.0486499 | M300-M0 |
| TR33131\|c0_g1_i1\|m.15228 | dynamin-related 1C-like isoform 2 | membrane | Cellular component | GO:0016020 | Non-expressed | 0.0486499 | M300-M0 |
| TR26801\|c0_g1_i1\|m.12368 | Phospho-2-dehydro-3-deoxyheptonate aldolase chloroplast | membrane | Cellular component | GO:0016020 | Non-expressed | 0.0486499 | M300-M0 |
| TR160004\|c0_g1_i1\|m.107220 | HVA22 f-like | membrane | Cellular component | GO:0016020 | Non-expressed | 0.0486499 | M300-M0 |
| TR141777\|c0_g3_i1\|m.98610 | Alpha-soluble NSF attachment | membrane | Cellular component | GO:0016020 | Non-expressed | 0.0486499 | M300-M0 |
| TR136395\|c0_g1_i1\|m.94207 | 40S ribosomal S9 | membrane | Cellular component | GO:0016020 | Non-expressed | 0.0401827 | M300-M0 |
| TR120037\|c0_g1_i1\|m.79357 | developmentally regulated GTP binding 1 | membrane | Cellular component | GO:0016020 | Non-expressed | 0.0401827 | M300-M0 |
| TR113592\|c0_g1_i1\|m.73613 | chlorophyll a-b binding chloroplastic | membrane | Cellular component | GO:0016020 | Non-expressed | 0.0239981 | M300-M0 |
| TR119981\|c0_g1_i3\|m.79309 | CBS domain-containing CBSCBSPB3-like | membrane | Cellular component | GO:0016020 | Non-expressed | 0.0293628 | M300-M0 |
| TR98171\|c0_g1_i1\|m.59032 | thioredoxin 4A-like | nucleus | Cellular component | GO:0005634 | Non-expressed | 0.0293628 | M300-M0 |
| TR26416\|c0_g2_i1\|m.12182 | Importin ALPHA,AIMP ALPHA isoform 2 | nucleus | Cellular component | GO:0005634 | Non-expressed | 0.0293628 | M300-M0 |
| TR128920\|c0_g1_i3\|m.87434 | PPPDE peptidase domain-containing | nucleus | Cellular component | GO:0005634 | Non-expressed | 0.0293628 | M300-M0 |
| TR127978\|c0_g1_i1\|m.86394 | crooked neck 1 | nucleus | Cellular component | GO:0005634 | Non-expressed | 0.0272254 | M300-M0 |
| TR124599\|c0_g1_i1\|m.83925 | 26S proteasome non-ATPase regulatory subunit 2 partial | nucleus | Cellular component | GO:0005634 | Non-expressed | 0.0272254 | M300-M0 |
| TR101386\|c1_g2_i3\|m.62175 | SUMO-activating enzyme subunit 1B-1-like | nucleus | Cellular component | GO:0005634 | Non-expressed | 0.0272254 | M300-M0 |
| TR82919\|c2_g5_i10\|m.47782 | nadp-specific glutamate dehydrogenase | catalytic activity | Molecular function | GO:0003824 | Non-expressed | 0.0486499 | M300-M0 |
| TR74536\|c0_g1_i3\|m.39876 | beta-amyrin 28-oxidase-like | catalytic activity | Molecular function | GO:0003824 | Non-expressed | 0.0486499 | M300-M0 |
| TR139389\|c1_g1_i1\|m.96583 | 4-hydroxy-3-methylbut-2-enyl diphosphate chloroplastic | catalytic activity | Molecular function | GO:0003824 | Non-expressed | 0.0486499 | M300-M0 |
| TR138640\|c0_g2_i1\|m.95764 | cytochrome P450 71D10-like | catalytic activity | Molecular function | GO:0003824 | Non-expressed | 0.0486499 | M300-M0 |
| TR128954\|c2_g1_i1\|m.87498 | 6-phosphogluconate dehydrogenase | catalytic activity | Molecular function | GO:0003824 | Non-expressed | 0.0486499 | M300-M0 |
| TR81947\|c0_g1_i1\|m.46080 | caffeic acid 3-O-methyltransferase 1-like isoform X2 | transferase activity | Molecular function | GO:0016740 | Non-expressed | 0.0486499 | M300-M0 |
| TR50407\|c0_g1_i1\|m.23142 | UDP-glucose flavonoid 3-O-glucosyltransferase 7-like | transferase activity | Molecular function | GO:0016740 | Non-expressed | 0.0486499 | M300-M0 |
| TR142640\|c0_g1_i1\|m.99551 | BAHD acyltransferase At5g47980-like | transferase activity | Molecular function | GO:0016740 | Non-expressed | 0.0486499 | M300-M0 |
| TR92759\|c8_g8_i3\|m.55244 | translation elongation factor 1a | kinase activity | Molecular function | GO:0016301 | Non-expressed | 0.0486499 | M300-NM300 |
| TR73873\|c0_g1_i2\|m.39195 | ribose-phosphate pyrophosphokinase 4 | kinase activity | Molecular function | GO:0016301 | Non-expressed | 0.0486499 | M300-NM300 |
| TR109719\|c0_g1_i4\|m.70579 | inositol polyphosphate multikinase beta-like | kinase activity | Molecular function | GO:0016301 | Non-expressed | 0.0401827 | M300-NM300 |
| TR141539\|c0_g1_i1\|m.98395 | cytochrome b-c1 complex subunit 7-2-like | mitochondrion | Cellular component | GO:0005739 | Non-expressed | 0.0401827 | M300-NM300 |
| TR85343\|c0_g1_i1\|m.49167 | glycerol-3-phosphate dehydrogenase mitochondrial-like | mitochondrion | Cellular component | GO:0005739 | Non-expressed | 0.0043405 | M300-NM300 |
| TR81698\|c0_g1_i1\|m.45812 | PREDICTED: uncharacterized protein C23H3.12c-like | mitochondrion | Cellular component | GO:0005739 | Non-expressed | 0.0239981 | M300-NM300 |
| TR61414\|c0_g1_i1\|m.29124 | cytochrome c oxidase subunit 6b-2-like | mitochondrion | Cellular component | GO:0005739 | Non-expressed | 0.0239981 | M300-NM300 |
| TR21072\|c0_g1_i1\|m.9482 | porin voltage-dependent anion-selective channel | mitochondrion | Cellular component | GO:0005739 | Non-expressed | 0.0239981 | M300-NM300 |
| TR120769\|c0_g1_i1\|m.79986 | AF407337_1Ras-related Rab7 | mitochondrion | Cellular component | GO:0005739 | Non-expressed | 0.0293628 | M300-NM300 |
| TR81224\|c0_g1_i2\|m.45342 | mitochondrial-like isoform X1 | membrane | Cellular component | GO:0016020 | Non-expressed | 0.0293628 | M300-NM300 |
| TR143173\|c0_g1_i1\|m.100249 | transmembrane 184C-like | membrane | Cellular component | GO:0016020 | Non-expressed | 0.0293628 | M300-NM300 |
| TR122742\|c0_g3_i1\|m.81650 | ATP-dependent Clp protease proteolytic subunit (chloroplast) | membrane | Cellular component | GO:0016020 | Non-expressed | 0.0293628 | M300-NM300 |
| TR115070\|c0_g2_i1\|m.75355 | syntaxin-121-like | membrane | Cellular component | GO:0016020 | Non-expressed | 0.0293628 | M300-NM300 |
| TR110867\|c0_g1_i1\|m.72420 | 60S ribosomal L23 | membrane | Cellular component | GO:0016020 | Non-expressed | 0.0293628 | M300-NM300 |
| TR109857\|c0_g2_i5\|m.70885 | cysteine-rich repeat secretory 38-like | membrane | Cellular component | GO:0016020 | Non-expressed | 0.0293628 | M300-NM300 |
